# Supplementary material for: Alcohol-related liver disease disrupts bile acid homeostasis and gut microbial bile acid metabolism
Source: JHEP Rep. 2026 Apr 2;8(7):101848. doi: 10.1016/j.jhepr.2026.101848 (PMC13233754; doi:10.1016/j.jhepr.2026.101848)
Supplement: Mumtimedia component 1 [file mmc1.pdf]

Alcohol-related liver disease disrupts bile acid homeostasis and gut microbial bile acid metabolism

Marisa Isabell Keller, Andressa de Zawadzki, Maja Thiele, Tommi Suvitaival, Karolina Sulek, Michael Kuhn, Christian Schudoma, Daniel Podlesny, Suguru Nishijima, Anthony Fullam, Chan Yeong Kim, Lili Niu, Asger Wretlind, Johanne Krag Hansen, Mads Israelsen, Stine Johansen, Wasiu Akanni, Diënty Hazenbrink, Helene Baek Juel, Matthias Mann, Torben Hansen, Aleksander Krag, Peer Bork, Cristina Legido-Quigley, on behalf of GALAXY & MicrobLiver consortia

Table of contents

Fig. S1 2
Fig. S2 3
Fig. S3 4
Fig. S4 5
Fig. S5 6
Fig. S6 7
Fig. S7 8
Fig. S8 9
Fig. S9 10
Fig. S10 11
Fig. S11 12
Table S1 13

A

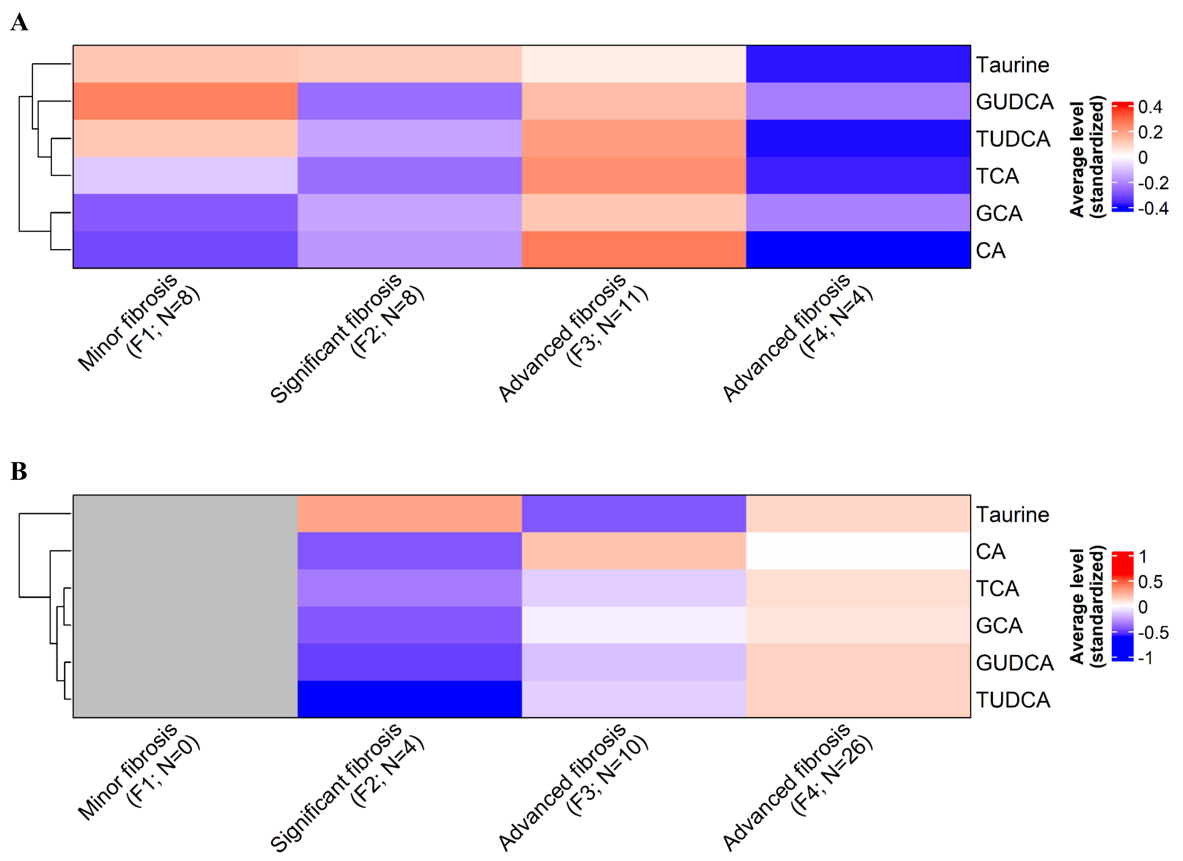

Fig. S1

Blood metabolite levels according to fibrosis stage in the validation cohorts 1 and 2 (A and B, respectively).

Metabolites are shown in rows (ordered by hierarchical clustering), fibrosis stages in columns, and the respective standardized average level in color (red: above cohort average; blue: below cohort average).

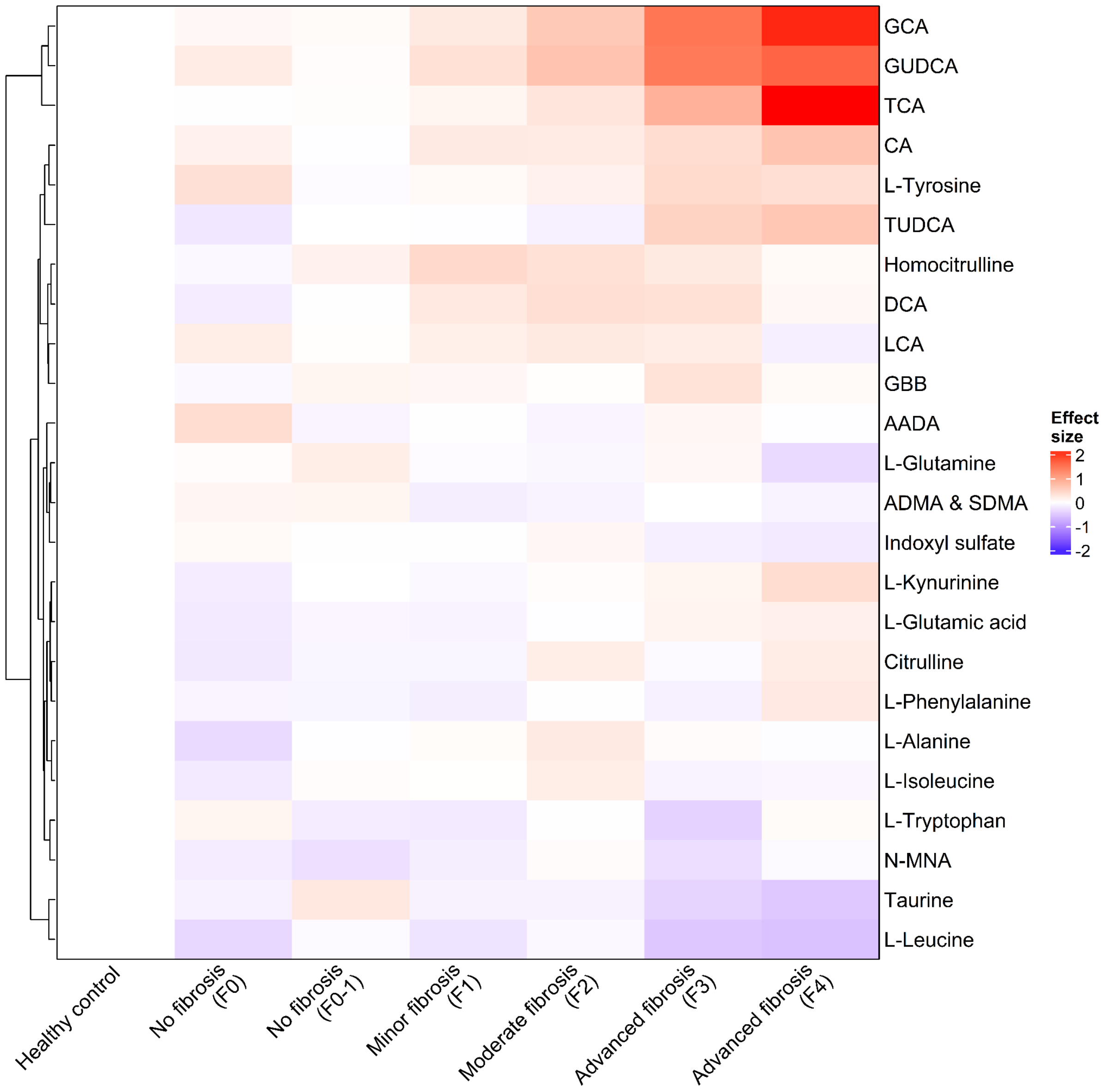

Fig. S2

Blood metabolite levels according to fibrosis stage. Heatmap of the standardized effect size of metabolite level as compared to healthy controls with all measured metabolites in blood. Metabolites are shown in rows (ordered by hierarchical clustering), fibrosis stages in columns, and the respective effect size in color (red: increase; blue: decrease). **Amino acids**: Citrulline, L-alanine, L-glutamic acid, L-glutamine, L-isoleucine, L-leucine, L-phenylalanine, L-tryptophan, L-tyrosine; **Amino acid derivatives**: Asymmetric dimethylarginine and symmetric dimethylarginine (ADMA & SDMA), homocitrulline, L-kynurinine, gamma-butyrobetaine (GBB), taurine; **Primary bile acids**: Cholic acid (CA), chenodeoxycholic acid (CDCA); **Secondary bile acids**: Deoxycholic acid (DCA), lithocholic acid (LCA); **Conjugated primary bile acids**: glycocholic acid (GCA), glycochenodeoxycholic acid (GCDCA), taurocholic acid (TCA), taurochenodeoxycholic acid (TCDCA);

Conjugated secondary bile acids: Glycodeoxycholic acid (CDCA), glycolithocholic acid (GLCA), glycoursodeoxycholic acid (GUDCA), taurodeoxycholic acid (TDCA), tauroursodeoxycholic acid (TUDCA); **Other compounds**: arylacetamide deacetylase (AADA), azelaic acid, N-1-methylnicotinamide (N-MNA), indoxyl sulfate

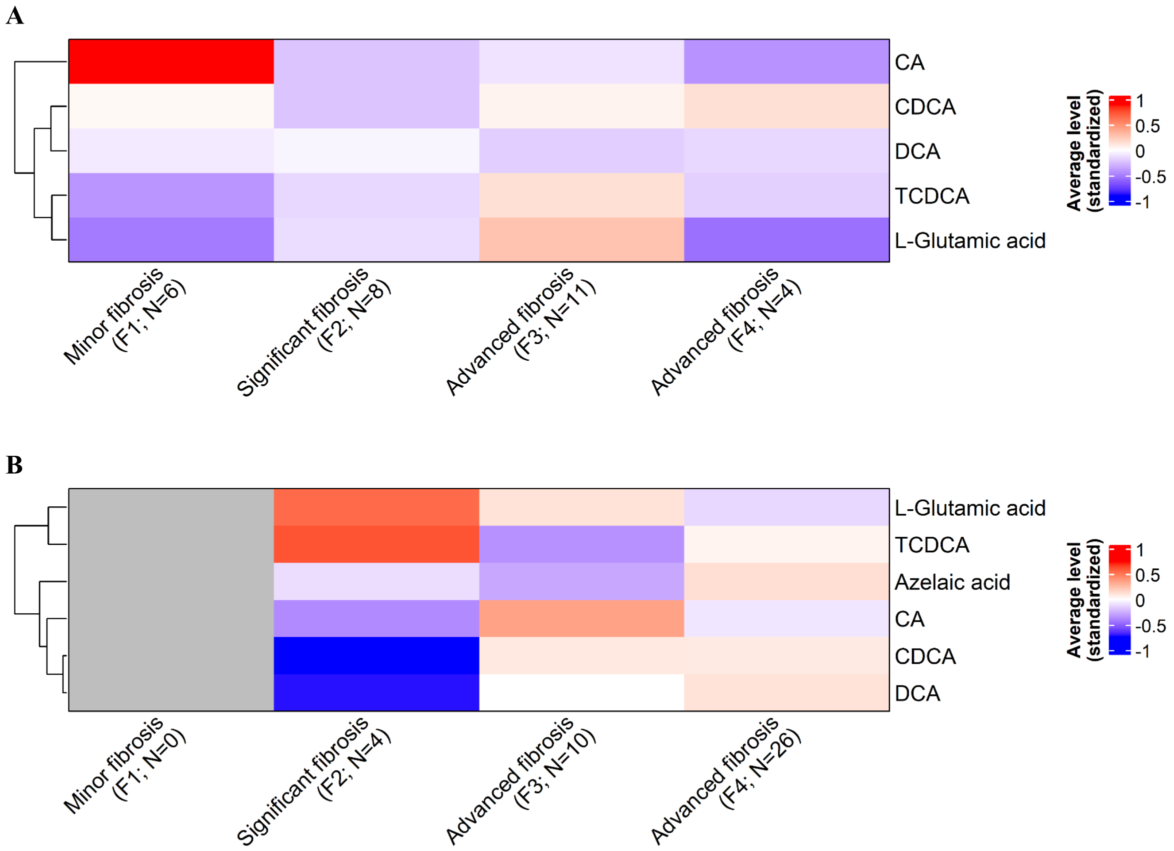

Fig. S3

Fecal metabolite levels according to fibrosis stage in the validation cohorts 1 and 2 (A and B, respectively). Metabolites are shown in rows (ordered by hierarchical clustering), fibrosis stages in columns, and the respective standardized average level in color (red: above cohort average; blue: below cohort average).

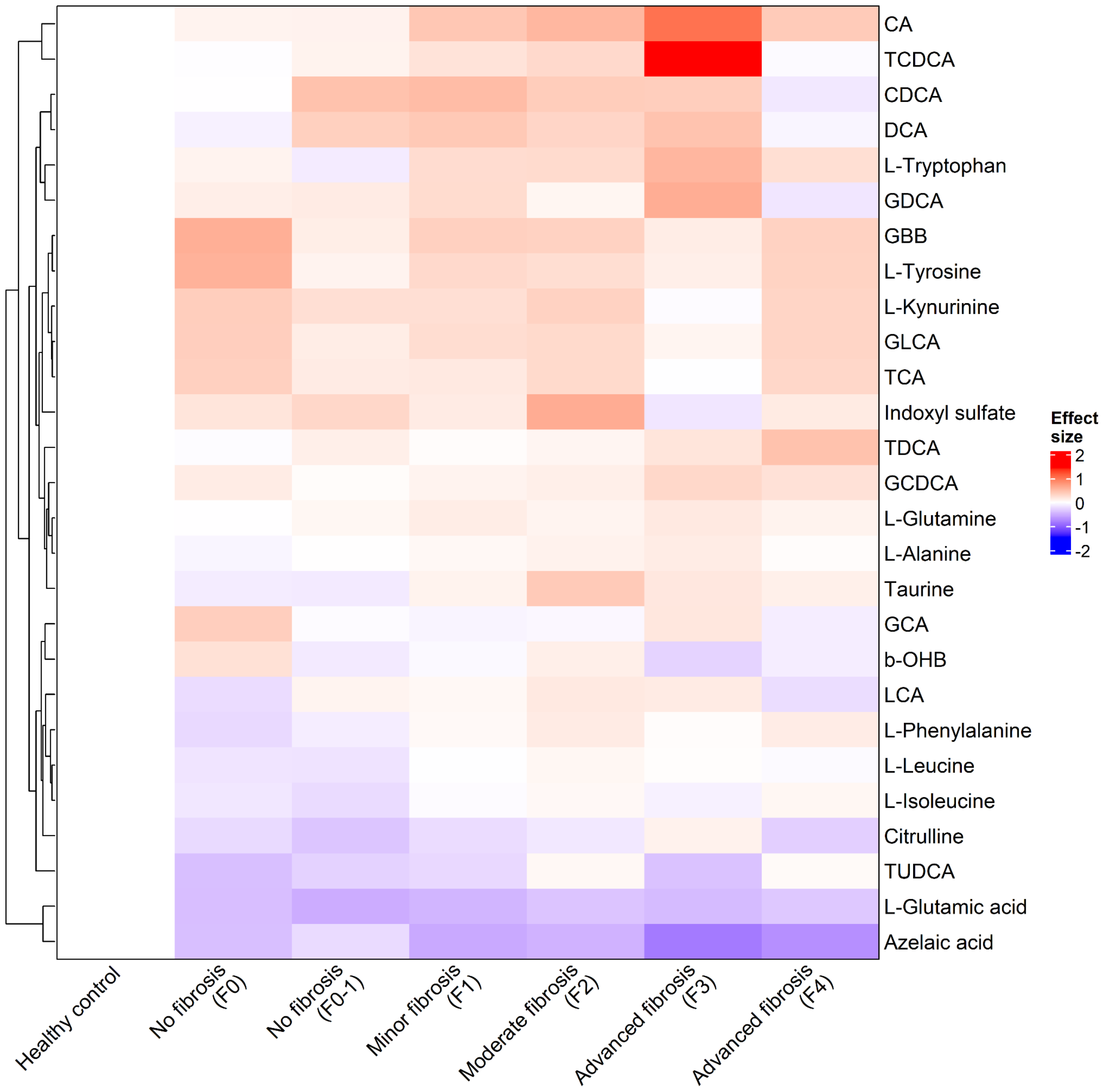

Fig. S4

Fecal metabolite levels according to fibrosis stage. Heatmap of the standardized effect size of metabolite level as compared to healthy controls with all measured metabolites in feces. Metabolites are shown in rows (ordered by hierarchical clustering), fibrosis stages in columns, and the respective effect size in color (red: increase; blue: decrease). **Amino acids**: Citrulline, L-alanine, L-glutamic acid, L-glutamine, L-isoleucine, L-leucine, L-phenylalanine, L-tryptophan, L-tyrosine; **Amino acid derivatives**: Asymmetric dimethylarginine and symmetric dimethylarginine (ADMA & SDMA), homocitrulline, L-kynurinine, gamma-butyrobetaine (GBB), taurine; **Primary bile acids**: Cholic acid (CA), chenodeoxycholic acid (CDCA); **Secondary bile acids**: Deoxycholic acid (DCA), lithocholic acid (LCA); **Conjugated primary bile acids**: glycocholic acid (GCA), glycochenodeoxycholic acid (GCDCA), taurocholic acid (TCA), taurochenodeoxycholic acid (TCDCA);

Conjugated secondary bile acids: Glycodeoxycholic acid (CDCA), glycolithocholic acid (GLCA), glycoursodeoxycholic acid (GUDCA), taurodeoxycholic acid (TDCA), tauroursodeoxycholic acid (TUDCA); **Other compounds**: arylacetamide deacetylase (AADA), azelaic acid, N-1-methylnicotinamide (N-MNA), indoxyl sulfate

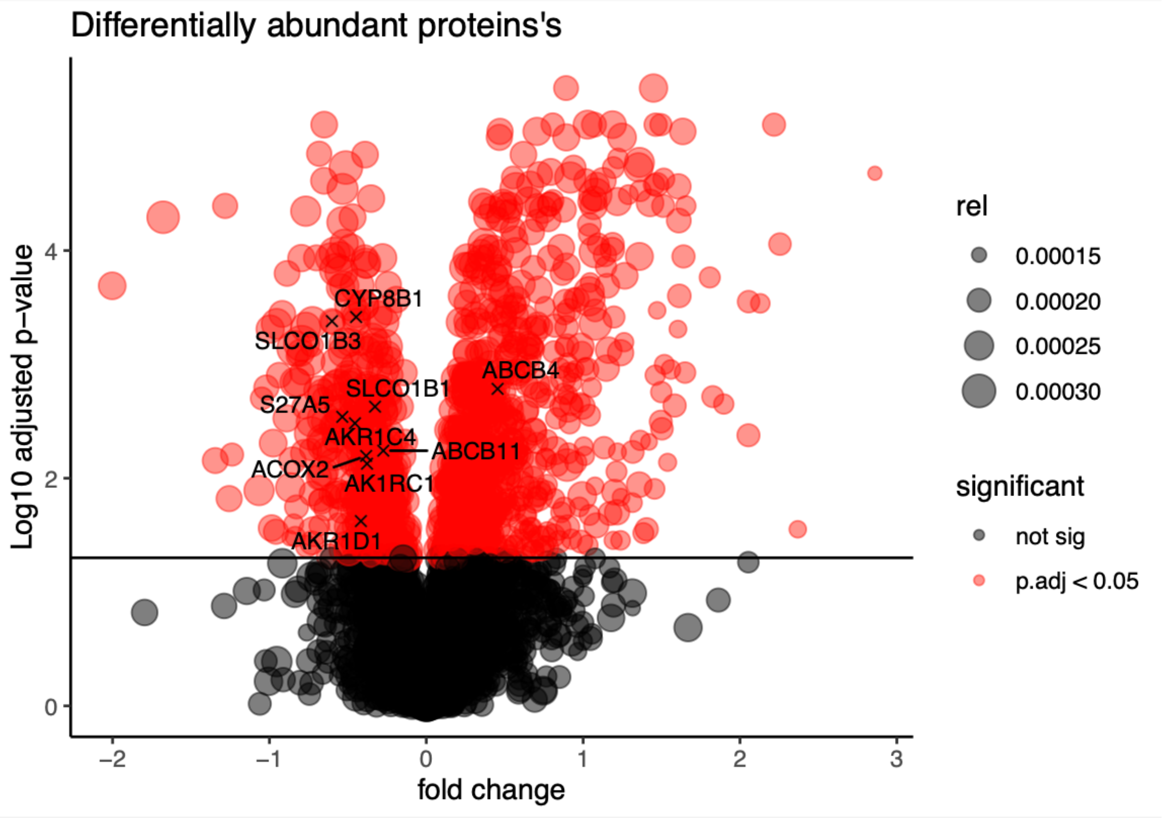

Fig. S5

Differential abundance analysis of the liver proteomics dataset comparing early fibrosis (F0-1) with late fibrosis (F2-4). Proteins in red are significantly expressed after correcting for false positives using the Benjamini-Hochberg correction. Points with labels are related to BA synthesis and transport.

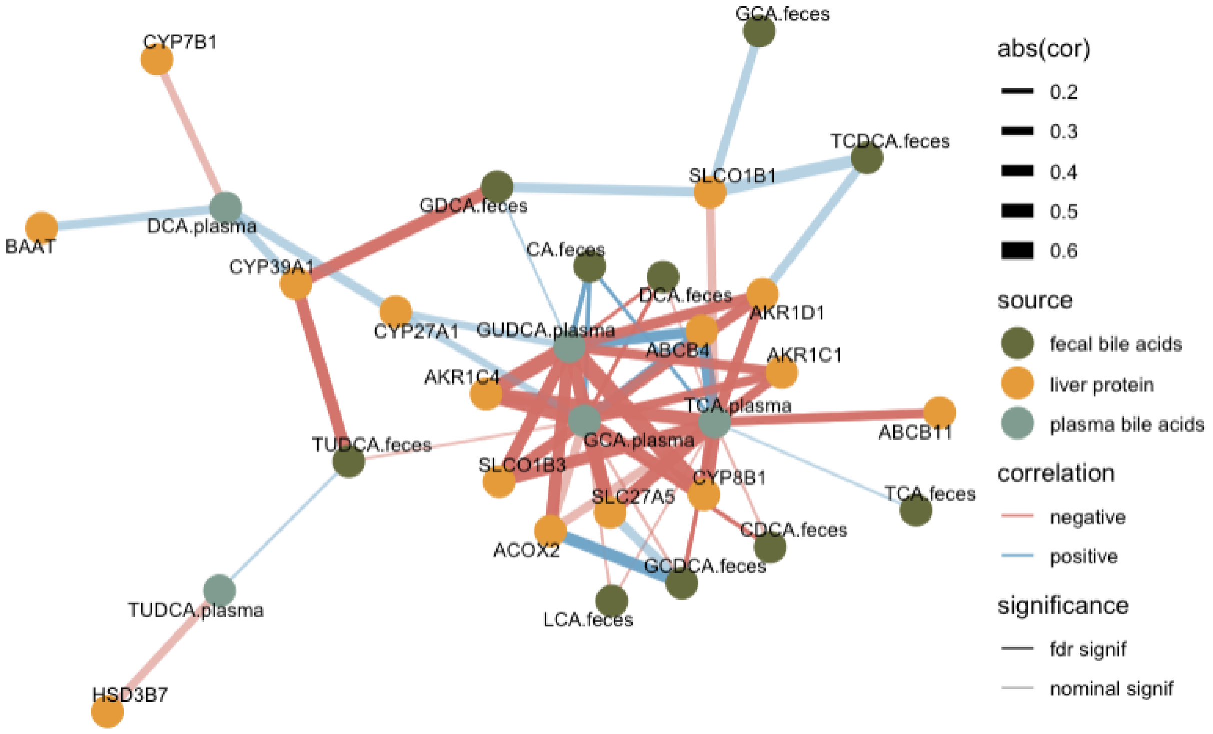

Fig. S6

Network analysis of liver proteins related to bile acid metabolism and bile acids measured in feces and plasma. The network is based on Spearman correlations, and "cor" represents the absolute value of the Spearman ρ. Shown are only the correlations with nominal significance and correlations with FDR-corrected significance, when correcting for all correlations performed in this study (including plasma bile acids, fecal bile acids, liver proteins, microbial abundance, and microbial gene abundances).

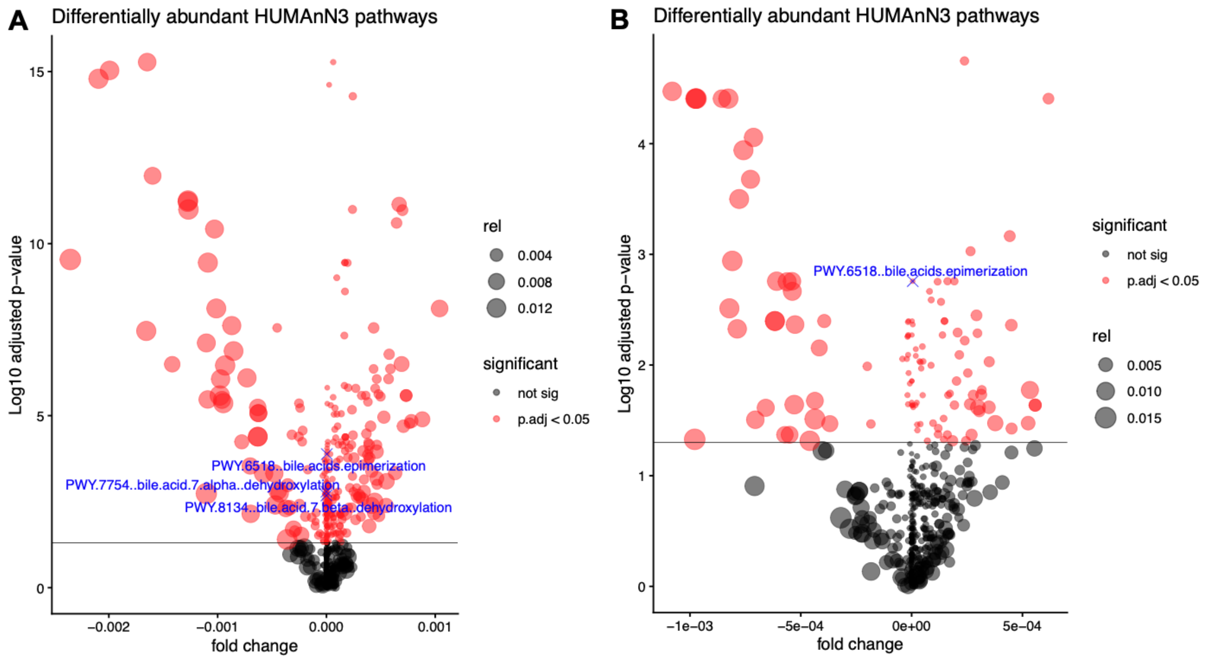

Fig. S7

Differential abundance analysis of the microbial pathways derived from metagenomic sequencing of fecal samples. The test compares the ALD with the HC samples (A) and within the ALD cohort, the early fibrosis (F0-1) with late fibrosis (F2-4) (B). Points in red are significantly changing pathways between the comparisons after adjusting for false positives using the Benjamini-Hochberg correction. Points with labels show secondary BA metabolism pathways.

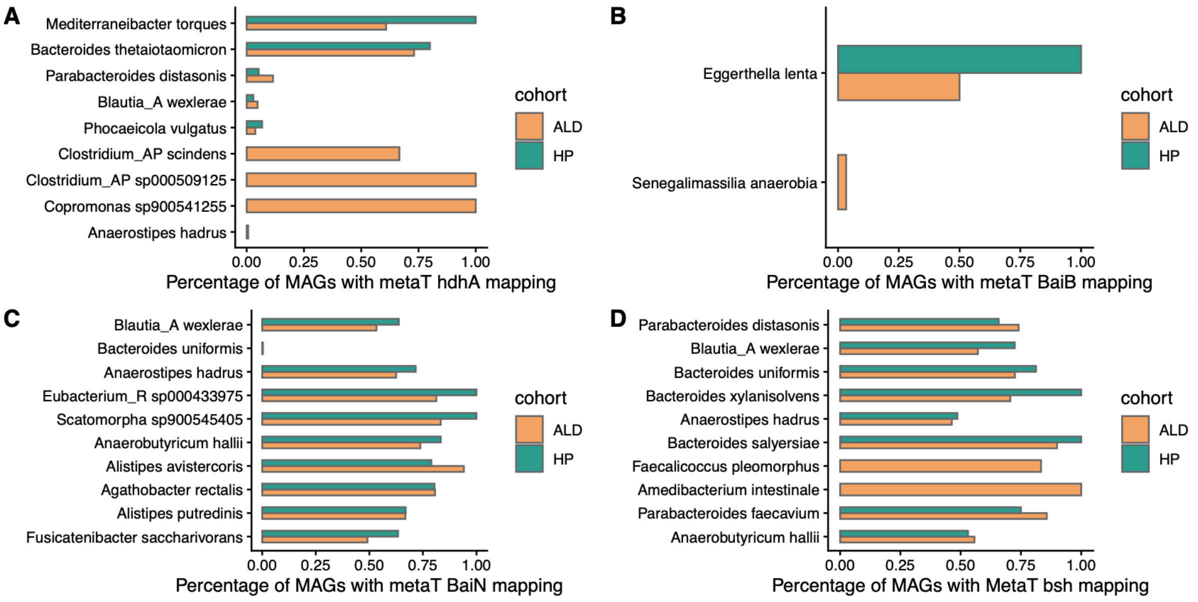

Fig. S8

Mapping of the metatranscriptomic reads assigned to the KEGG orthologs to the MAGs within the cohort. The fraction of MAGs is calculated by dividing the species-specific MAGs with mapping for this gene by the total amount of species-specific MAGs. The species are selected to correspond to the species reported in figure 5 and Fig. S6. hdhA = K00076, BaiB = K15868, BaiN = K07007, bsh = K01442

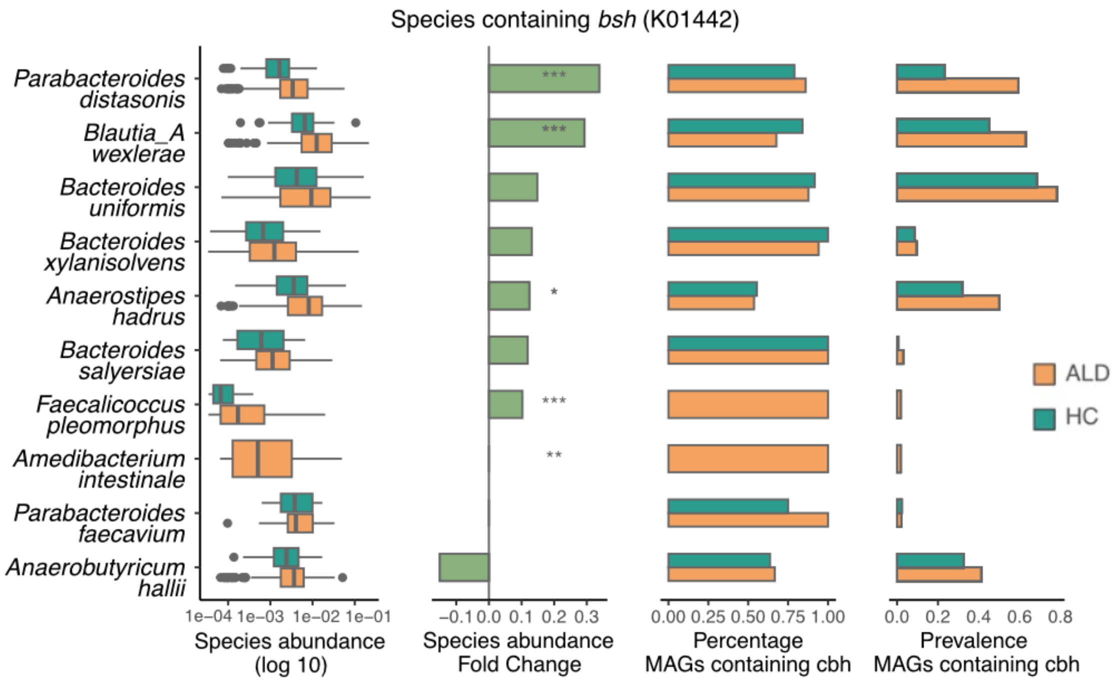

Fig. S9

Microbial taxa for the secondary bile acid metabolism deconjugation pathway facilitated by *bsh*. The species abundance, species fold change between healthy and ALD samples, the gene-carrying frequency of MAGs, and the prevalence of gene-carrying MAGs in the cohort collectively estimate the importance of the taxa in ALD.

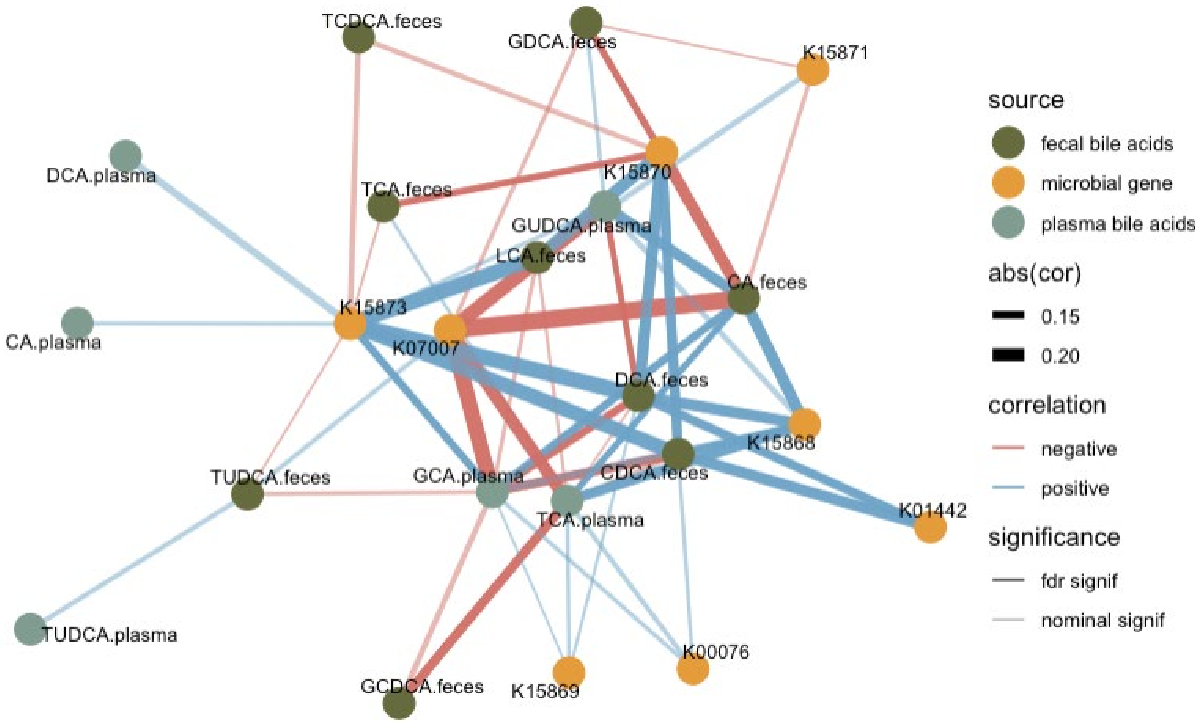

Fig. S10

Network analysis of microbial bile-acid metabolism-related KOs and bile acids measured in feces and plasma. The network is based on Spearman correlations, and "cor" represents the absolute value of the Spearman ρ. Shown are only the correlations with nominal significance and correlations with FDR-corrected significance, when correcting for all correlations performed in this study (including plasma bile acids, fecal bile acids, liver proteins, microbial abundance, and microbial gene abundances). K07007 = BaiN, K00076 = hdhA, K01442 = bile-salt-hydrolase (bsh), K15868 = BaiB, K15869 = BaiA, K15870 = BaiCD, K15871 = BaiF, K15873 = BaiH.

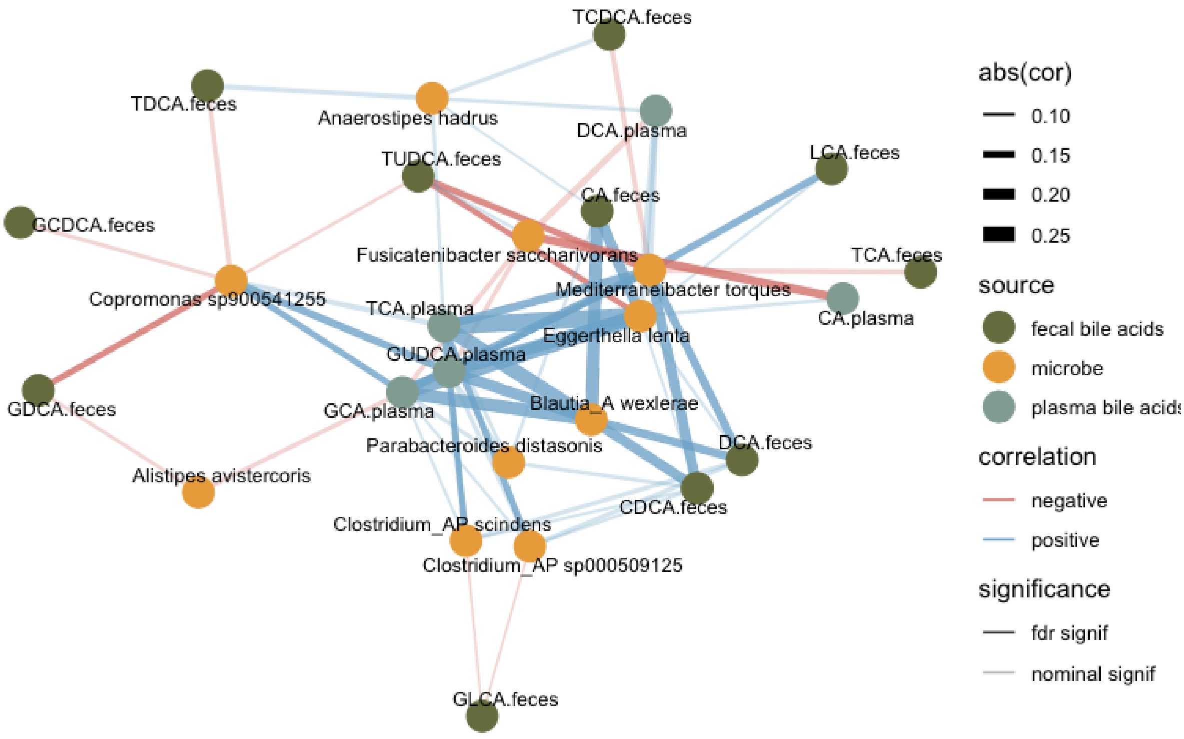

Fig. S11

Network analysis of microbial abundances and bile acids measured in feces and plasma. The network is based on Spearman correlations and “cor” represents the absolute value of the Spearman ρ. Shown are only the correlations with nominal significance and correlations with FDR-corrected significance, when correcting for all correlations performed in this study (including plasma bile acids, fecal bile acids, liver proteins, microbial abundance, and microbial gene abundances). Shown are only the microbes that we identified as relevant in our study based on the gene mappings to their MAGs. But all microbes with a mean abundance higher than 1e-4 and a prevalence of more than 10% of the samples have been included in the correlation analysis.

	GALA-ALD	Validation cohort 1	Validation cohort 2	p-value
**Characteristics**				
Participants (n)	462	34	52	NA
Female sex (n, %)	112(24.2%)	2(5.9%)	14(26.9%)	0.0371
Age (years)	56.5 ± 10.4	59.2 ± 8.7	60.7 ± 7.9	0.0122
BMI (kg/m^2)	27.5 ± 5.3	30.5 ± 5.3	29 ± 5.4	0.0006
T2D (n, %)	64(13.9%)	0(0%)	2(3.8%)	0.0431
Metabolic syndrome (n, %)	114(24.7%)	14(41.2%)	20(38.5%)	0.0650
Abstinent (n, %)	193(41.8%)	30(88.2%)	17(32.7%)	0.0000
**Liver parameter**				
Cap Value (dB/m)	284.8 ± 61.7	306.4 ± 61	288.8 ± 67.4	0.0000
Kleiner Score (0/0-1/1/2/3/4)	36/98/127/107/27/66	0/0/8/8/11/4	0/0/0/5/10/29	0.0000
Bilirubin (umol/L)	12.6 ± 9.5	9 ± 4.7	15.8 ± 9.5	0.0002
ALT (U/L)	39.4 ± 29.5	46.5 ± 41	33.7 ± 30.8	0.0089
AST (U/L)	45.5 ± 35.2	NaN ± NA	42.4 ± 25	0.9551
GGT (U/L)	182.6 ± 315.8	162.9 ± 155.7	180 ± 183.7	0.0515
Platelets (10^9/L)	237 ± 87.6	212.8 ± 51.5	168.1 ± 64	0.0000
MELD-score	7.1 ± 1.8	7.8 ± 1.5	8.7 ± 1.6	0.0000

Table S1

Characteristics of the GALAXY alcohol-related liver disease (ALD) cohort in comparison to the Validation 1 and 2 cohorts. T2D = Type 2 Diabetes, Cap = Controlled attenuation parameter, ALT = alanine transaminase, AST = aspartate aminotransferase, GGT = gamma-glutamyl transferase, MELD = model of end-stage liver disease. The continuous variables are presented as means with standard deviations. P-values report the significance of the differences between the ALD and HC cohorts tested by the Kruskal-Wallis test for continuous variables and the chi-squared test for categorical variables.
