# Supplementary material for: Alcohol-related liver disease disrupts bile acid homeostasis and gut microbial bile acid metabolism
Source: JHEP Rep. 2026 Apr 2;8(7):101848. doi: 10.1016/j.jhepr.2026.101848 (PMC13233754; doi:10.1016/j.jhepr.2026.101848)
Supplement: Mumtimedia component 2 [file mmc2.docx]

**Journal of Hepatology**

**CTAT methods**

Tables for a “Complete, Transparent, Accurate and Timely account” (CTAT) are now mandatory for all revised submissions. The aim is to enhance the reproducibility of methods.

- Only include the parts relevant to your study
- Refer to the CTAT in the main text as ‘Supplementary CTAT Table’
- Do not add subheadings
- Add as many rows as needed to include all information
- Only include one item per row

**If the CTAT form is not relevant to your study, please outline the reasons why:**

|  |
| --- |

- 1. **Antibodies**

| **Name** | **Citation** | **Supplier** | **Cat no.** | **Clone no.** |
| --- | --- | --- | --- | --- |
|  |  |  |  |  |

- 1. **Cell lines**

| **Name** | **Citation** | **Supplier** | **Cat no.** | **Passage no.** | **Authentication test method** |
| --- | --- | --- | --- | --- | --- |
|  |  |  |  |  |  |

- 1. **Organisms**

| **Name** | **Citation** | **Supplier** | **Strain** | **Sex** | **Age** | **Overall n number** |
| --- | --- | --- | --- | --- | --- | --- |
|  |  |  |  |  |  |  |

- 1. **Sequence based reagents**

| **Name** | **Sequence** | **Supplier** |
| --- | --- | --- |
|  |  |  |

- 1. **Biological samples**

| **Description** | **Source** | **Identifier** |
| --- | --- | --- |
|  |  |  |

- 1. **Deposited data**

| **Name of repository** | **Identifier** | **Link** |
| --- | --- | --- |
|  |  |  |

- 1. **Software**

| **Software name** | **Manufacturer** | **Version** |
| --- | --- | --- |
| ngless | https://ngless.embl.de/ | V1.1 |
| mOTUs | https://motu-tool.org/ | V2.5 |
| GTDB-tk | https://github.com/Ecogenomics/GTDBTk | V2.11 r207 |
| proGenomes | https://progenomes.embl.de/ |  |
| Global microbial gene catalog (GMGC) | https://gmgc.embl.de/ |  |
| BWA-MEM | https://maq.sourceforge.net/ | V0.7.17 |
| eggNOG-mapper | http://eggnog-mapper.embl.de/ | V1.0.3 |
| eggnog database | http://eggnog5.embl.de/#/app/home | 5.0 |
| Gffquant | <https://github.com/cschu/gff_quantifier> |  |
| Rstudio | www.r-studio.com | R 4.3.2 |
| Limma R-package | Bioconductor |  |
| Spectronaut | Biognosys | 15.4 and 13 |
| MassHunter Quantitative Analysis | Agilent Technologies |  |

- 1. **Other (e.g. drugs, proteins, vectors etc.)**

| **Name** | **Manufacturer** | **ID** |
| --- | --- | --- |
| All Prep Power Fecal DNA/RNA Kit | Qiagen | Catalog no. 80244 |
| NEBNext Bacteria rRNA Depletion Kit | New England Biolabs, Ipswich, Massachusetts, USA |  |
| NEBNext Ultra II DNA library kit | New England Biolabs, Ipswich, Massachusetts, USA |  |
| NEBNext Ultra II Directional RNA Library Prep Kit | New England Biolabs, Ipswich, Massachusetts, USA |  |
| Illumina HiSeq 4000 platform | Illumina, San Diego, CA, USA |  |
| Proteomics lysis buffer | PreOmics, Planegg, Germany |  |
| Isopropanol | Fischer Scientific, Pennsylvania, USA |  |
| Trifluoroacetic acid | Sigma Aldrich, Massachusetts, USA |  |
| Acetonitrile | Fischer Scientific, Massachusetts, USA |  |
| LC-MS formic acid | Fischer Scientific, Massachusetts, USA |  |
| Q Exactive HF-X Orbitrap mass spectrometer | Thermo Fischer Scientific, Massachusetts, USA |  |
| EASY nLC-1200 system | Thermo Fischer Scientific,  Massachusetts, USA |  |
| cryoPREP Dry Pulverizer | Covaris, Massachusetts, USA |  |
| Diagenode Bioruptor | Diagenode, Seraing, Belgium |  |
| Adaptive Focused Acoustic sonication system | Covaris, Massachusetts, USA |  |
| SpeedVac Centrifuge | Eppendorf, Hamburg, Germany |  |
| Thermo mixer | Eppendorf, Hamburg, Germany |  |
| Nanodrop Spectrophotometer | Thermo Fischer Scientific,  Massachusetts, USA |  |

- 1. **Please provide the details of the corresponding methods author for the manuscript:**

|  |
| --- |

**2.0 Please confirm for randomised controlled trials all versions of the clinical protocol are included in the submission. These will be published online as supplementary information.**

| GALAXY discovery cohort: Danish Data Protection Agency nos. 13/8204, 16/3492 and 18/22692; and Odense Patient Data Exploratory Network under study identification nos. OP_040 and OP_239  Validation cohort 1: EudraCT number 20214-001856-51  Validation cohort 2: ClinicalTrial.gov ID NCT03863730 |
| --- |
