# Supplementary material for: Alcohol-related liver disease disrupts bile acid homeostasis and gut microbial bile acid metabolism
Source: JHEP Rep. 2026 Apr 2;8(7):101848. doi: 10.1016/j.jhepr.2026.101848 (PMC13233754; doi:10.1016/j.jhepr.2026.101848)
Supplement: Mumtimedia component 5 [file mmc5.pdf]

JHEP|Reports

Alcohol-related liver disease disrupts bile acid homeostasis and gut microbial bile acid metabolism

Authors

Marisa Isabell Keller, Andressa de Zawadzki, Maja Thiele, …, Torben Hansen, Aleksander Krag, Peer Bork§Cristina Legido-Quigley

Correspondence

cristina.legido_quigley@kcl.ac.uk (C. Legido-Quigley).

Graphical abstract

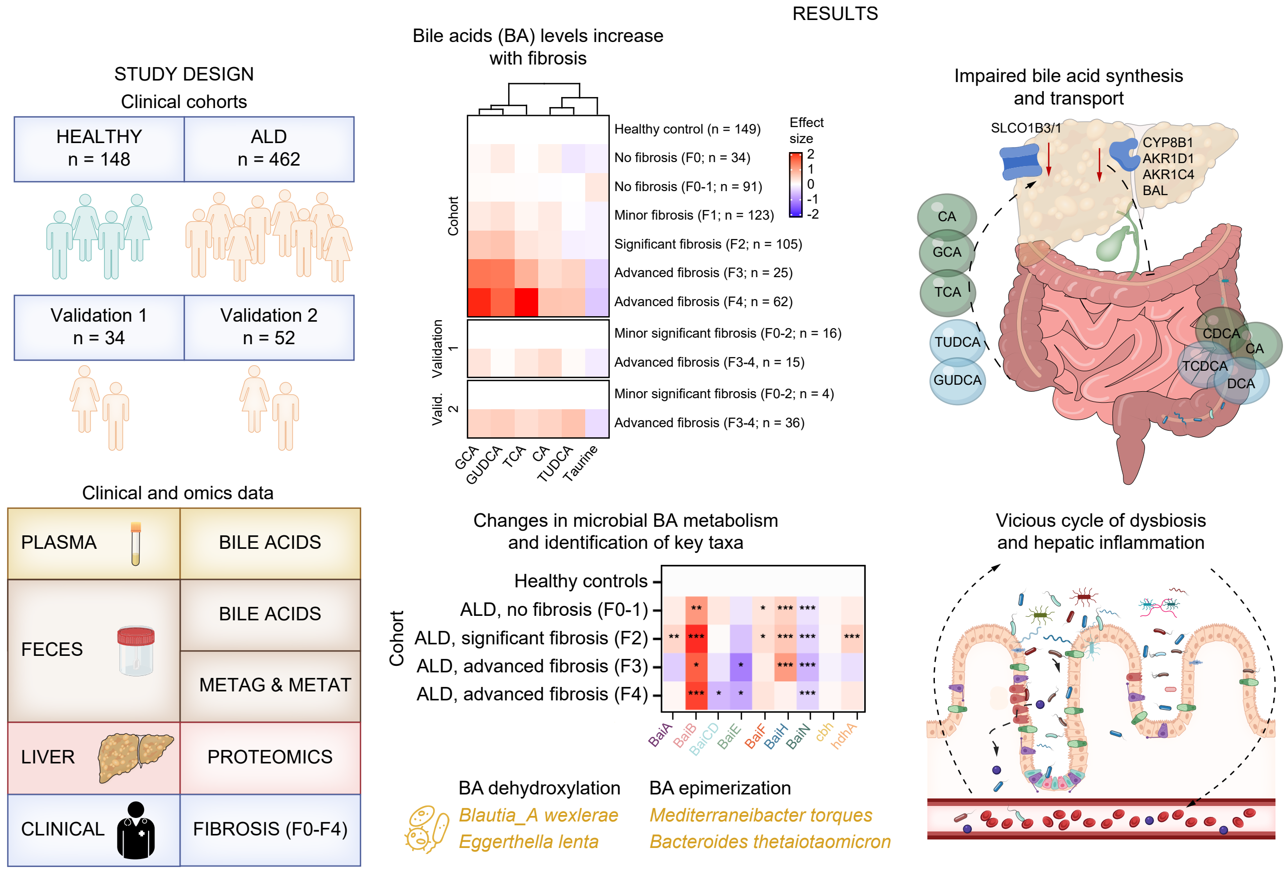

Highlights:

- Bile acids in serum and feces increase with progression of alcohol-related liver disease.
- Bile acid production and uptake from the blood are impaired in the liver.
- Secondary microbial bile acid metabolism, particularly epimerization and dehydroxylation, increases in alcohol-related liver disease.
- However, the key enzyme, *baiN,* is depleted, illustrating a microbial community-level metabolic dysbiosis.
- *M. torques*, *B. thetaiotaomicron*, and *Blautia_A wexlerae* could serve as potential targets for future interventions.

Impact and implications:

This study shows that integrating different omics approaches provides insight into metabolic disruptions across the gut–liver axis that drive ALD progression. Additionally, our study identifies specific bacterial species influencing BA concentrations in ALD using data from human fecal metagenomics and metatranscriptomics. These findings could inform the design of future therapeutic targets focusing on either the liver or the gut for treating ALD.

https://doi.org/10.1016/j.jhepr.2026.101848
© 2026 The Authors. Published by Elsevier B.V. on behalf of European Association for the Study of the Liver (EASL). This is an open access article under the CC BY license (http://creativecommons.org/licenses/by/4.0/).

Alcohol-related liver disease disrupts bile acid homeostasis and gut microbial bile acid metabolism

Marisa Isabell Keller[1,†], **Andressa de Zawadzki**[2,†], Maja Thiele[3,4], Tommi Suvitaival[2], Karolina Sulek[2,5], Michael Kuhn[1], Christian Schudoma[1], Daniel Podlesny[1], Suguru Nishijima[1], Anthony Fullam[1], Chan Yeong Kim[1], Lili Niu[6], Asger Wretlind[2], Johanne Krag Hansen[3,4], Mads Israelsen[3,4], Stine Johansen[3,4], Wasiu Akanni[1], Diënty Hazenbrink[1], Helene Baek Juel[7], Matthias Mann[6,8], Torben Hansen[7], Aleksander Krag[3,4], Peer Bork[1,9,10,‡,§], Cristina Legido-Quigley[2,11,*,‡], on behalf of GALAXY & MicrobLiver consortia[#]

JHEP Reports **2026**. vol. 8 | 1–14

Check for updates

Background & Aims: Alcohol overuse disrupts liver function and alters gut microbial communities, with alcohol-related liver disease (ALD) causing half of all liver-related deaths worldwide. Bile acids (BAs) regulate liver and gut function, but their homeostasis becomes disrupted in ALD. Gut microbes transform primary BAs to secondary BAs, which are reabsorbed via enterohepatic circulation, but BA metabolism during ALD progression remains poorly understood.

Methods: We investigated BA homeostasis in a cross-sectional ALD cohort (n = 462), alongside matched healthy controls (n = 148), and validated key findings in two independent ALD cohorts (n = 34 and n = 52). We integrated BA concentrations, measured by targeted mass spectrometry in feces and plasma, with liver proteomics and gut microbiome profiles from metagenomic and metatranscriptomic sequencing.

Results: Advanced fibrosis states were associated with decreased hepatic BA synthesis, impaired hepatic BA uptake from blood but with increased levels of primary and secondary BAs in plasma (*inprimis*, taurocholic acid: $F = 69.9$, $p = 8.6e\text{-}66$) and feces (*inprimis*, cholic acid: $F = 5.5$, $p = 1.4e\text{-}4$). The abundance of microbial secondary BA dehydroxylation and epimerization pathways in the gut microbiome community increased with disease severity. Genes encoding the oxidation arm in the multistep dehydroxylation pathway (including baiB) increased, whereas those in the reduction arm (baiN) were depleted. In patients with ALD, we suggest *Eggerthella lenta, Mediterraneibacter torques*, and *Bacteroides thetaiotaomicron* as relevant microbes for BA metabolism.

Conclusion: Fibrotic ALD is characterized by disrupted primary BA synthesis and hepatic uptake, leading to hepatotoxic BA accumulation in the gut and blood circulation. Altered microbial secondary BA metabolism reflects a functional shift in the gut microbiome throughout the fibrosis stages. Our findings highlight the gut–liver axis as an important factor influencing ALD progression, even in early, asymptomatic fibrosis stages.

© 2026 The Authors. Published by Elsevier B.V. on behalf of European Association for the Study of the Liver (EASL). This is an open access article under the CC BY license (http://creativecommons.org/licenses/by/4.0/).

Introduction

Alcohol-related liver disease (ALD) causes significant changes in the abundance of molecules produced or metabolized by the liver.[1] Among those molecules, bile acids (BAs) have been shown to undergo significant metabolic changes following the progression of ALD and other liver diseases such as cholestatic diseases,[2] providing mechanistic insights into the disease development.[3] BA homeostasis protects the liver and other tissues from BA toxicity.[4] This enterohepatic circulation balances BA synthesis and transport across different compartments and contributes to cellular homeostasis.[5] Disruption of this equilibrium results in abnormally high BA concentrations that are cytotoxic.[6] Hydrophobic BAs act as detergents at elevated levels, causing misfolding of cytosolic proteins,[7] disrupting cellular membranes,[8] and inducing oxidative stress and DNA damage,[9] thereby leading to hepatocyte injury, biliary duct damage, inflammation, cholestasis, and liver fibrosis.

The liver and the intestinal microbiome collaborate in digestion through biochemical pathways involving fat metabolism, vitamin production, carbohydrate digestion, and detoxification.[10] Consequently, ALD-related metabolic changes have systemic effects and can be assessed via metabolite profiles in circulatory and excretory systems. BAs are synthesized by the liver from cholesterol and subsequently transported to the intestine, where they play a crucial role in fat digestion and vitamin absorption. Gut microbes convert primary BAs into secondary forms, which are recycled by the

* Corresponding author. Address: Franklin-Wilkins Building, 150 Stamford Street, London, SE1 9NH, UK.
E-mail address: cristina.legido_quigley@kcl.ac.uk (C. Legido-Quigley).
† These authors contributed equally.
‡ These authors contributed equally.
§ Deceased.
# GALAXY & MicrobLiver consortia members listed in Acknowledgements section.
https://doi.org/10.1016/j.jhepr.2026.101848

liver.[11] In the distal ileum, specific BA transporters reclaim up to 95% of the BAs, returning them to the liver via the portal vein, while ~5% are excreted in feces.[12]

Alcohol overuse in ALD is associated with alterations in the intestinal microbiome, which impact host metabolism.[13] As primary BA production is regulated by receptors in the gut epithelium,[14] gut microbes can inhibit BA production by modulating the expression of the 7-alpha-hydroxylase, the rate-limiting enzyme in BA synthesis.[15] Moreover, microbial secondary BA metabolism, including (re)conjugation with taurine and glycine, dehydrogenation, epimerization, and dehydroxylation, alters BA toxicity and intestinal permeability.[16]

Although studies have measured BAs using liquid chromatography and mass spectrometry, they often failed to integrate host and microbial BA metabolism.[17] Prior work linking 16S rRNA gene-based microbiome profiles to secondary BA metabolism capacity relied on correlations or species-specific *in vitro* studies, frequently limited to culturable bacteria.[18–20] However, bacterial metabolism can be environment-dependent and differ between *in vivo* and *in vitro* study conditions.[21] Thus, correlations between bacterial abundances and BAs may reflect indirect effects. Although the presence of specific genes and transcripts provides insight into BA metabolism, a gap remains in understanding which bacteria actively drive these processes.

Here, we aim to disentangle the contribution of microbial secondary BA metabolism to disrupted BA homeostasis in ALD. We integrated liver proteomics, serum and fecal targeted metabolomics, and fecal metagenomic and metatranscriptomic analyses to obtain a holistic view. We identify potential microbial species as biomarkers candidates and targets for microbial-based interventions, we mapped Kyoto Encyclopedia of Genes and Genomes (KEGG) orthologs to metagenome-assembled genomes (MAGs) from the same cohort, linking functional changes to specific microbial species by considering gene presence and prevalence within species.

Materials and methods

Cohort description

Main cohort

This study was conducted using plasma and fecal samples from the GALAXY cross-sectional cohort of individuals with a history of harmful drinking (men >36 g/day, women >24 g/day EtOH for at least 1 year) with an average of 19 years of excess alcohol use.[22–24] This cohort focuses on asymptomatic ALD across the early disease stages, excluding individuals with diagnosed chronic liver disease or evident signs of advanced, decompensated liver disease. Together with matched healthy controls (HCs) in the GALAXY-HC cohort (alcohol consumption units <7 units/week), the GALAXY-ALD cohort was used as the main discovery cohort. The HC group had normal glucose metabolism and liver function tests, no chronic or metabolic diseases, did not use antibiotics or any medications in the past 6 months, except occasional mild pain relievers. The Danish Data Protection Agency (13/8204, 16/3492) and the ethics committee for the Region of Southern Denmark (ethical ID S-20120071, S-20160021, S-20170087, and ID S-20160006G) approved these cohorts. Odense University Hospital recruited participants between 2013 and 2018, and obtained informed consent from all participants before inclusion. The study followed the ethical principles of the Declaration of Helsinki in all methods involving participants.

Validation cohorts

Baseline data from two additional ALD cohorts are used in this study, namely validation cohort 1[25] and validation cohort 2.[26] Validation cohort 1 is a randomized, single-center, placebo-controlled, double-blind study of patients with ALD with fibrosis F1–4 registered under EudraCT number 20214-001856-51. Validation cohort 2 is a randomized, controlled study of patients with ALD with compensated advanced chronic ALD (F3–4 and/or vibration-controlled transient elastography, VCTE, ≥10 kPa) registered in ClinicalTrial.gov ID NCT03863730.

Disease severity

We staged the participants with ALD from liver histology (biopsies with 17G Menghini suction needle; Hepafix, Braun, Germany) based on Kleiner fibrosis score[27] as F0 (no fibrosis), F1 (portal or periportal fibrosis), F2 (perisinusoidal fibrosis in combination with portal or periportal fibrosis), F3 (bridging fibrosis), and F4 (cirrhosis). For 97 participants from the GALAXY-ALD cohort, no liver histology was performed as their VCTE indicated no or minimal fibrosis (FibroScan <6.0 kPa; EchoSens, Paris, France). Consequently, those participants were grouped into the F0–F1 group in the analysis. The ALD and HC study populations differed in most liver health-related parameters, including liver stiffness measured, controlled attenuation parameter (CAP), and liver blood tests (Table 1). The samples from patients in the two validation cohorts showed similar ALD characteristics, but higher Kleiner scores, thus more advanced fibrosis (Table S1).

Targeted metabolomic dataset

Quantitative BA profiles were obtained from feces and plasma using a targeted method based on ultra-high-performance liquid chromatography coupled with a triple quadrupole mass spectrometer (ultra-high performance liquid chromatography-tandem mass spectrometry) as described previously.[28,29] This method targets BAs as well as key metabolites relevant to metabolic diseases.

Liver proteomics dataset

The liver proteome was acquired as described previously[22] and involved, in short, cryo-pulverizing liver biopsies, denaturing and digesting proteins with trypsin and LysC, acidifying to quench digestion, purifying peptides by solid-phase extraction, and drying them for concentration measurement. For LC–MS/MS analysis, 500 ng of purified peptides were injected and analyzed using data-independent acquisition on an EASY-nLC 1200 system with a Q Exactive HF-X Orbitrap, with subsequent data analysis conducted using Spectronaut software and stringent filtering for statistical robustness.

Metagenomic and metatranscriptomic of fecal samples

The metagenomic data were acquired as described previously;[23] metatranscriptomics sequences were acquired as described

Table 1. Characteristics of the GALAXY ALD and the GALAXY HC cohorts.

	GALA-ALD	GALA-HC	*p* values
**Characteristics**			
Participants (n)	462	148	NA
Female sex (n, %)	112 (24.2)	58 (39.2)	6.1e-04
Age (years)	56.5 ± 10.4	51.3 ± 12.2	3.2e-06
BMI (kg/m^2)	27.5 ± 5.3	26.4 ± 4.1	1.3e-02
T2D (n, %)	64 (13.9)	NA (NA)	NA
Metabolic syndrome (n, %)	114 (24.7)	NA (NA)	NA
Abstinent (n, %)	193 (41.8)	15 (10.1)	7.4e-17
Years of alcohol overuse	19.1 ± 11.4	NaN ± NA	NA
**Liver parameter**			
Liver stiffness/TE (kPa)	13.8 ± 17	4.6 ± 1.3	1.8e-23
CAP value (dB/m)	284.8 ± 61.7	251.8 ± 57.9	2.2e-07
Kleiner score (0/0–1/1/2/3/4)	36/98/127/107/27/66	0/0/0/0/0/0	2.8e-21
Bilirubin (μmol/L)	12.6 ± 9.5	11.4 ± 5.1	7.3e-01
ALT (U/L)	39.4 ± 29.5	25.7 ± 10.6	3.3e-09
AST (U/L)	45.5 ± 35.2	26.9 ± 11	1.3e-14
GGT (U/L)	182.6 ± 315.8	25.4 ± 15.6	2.2e-39
Platelets (10^9/L)	237 ± 87.6	237.9 ± 50	4.8e-01
MELD score	7.1 ± 1.8	6.6 ± 0.8	4.4e-02
CPA (mg/L)	5.8 ± 6.1	NaN ± NA	NA

The continuous variables are presented as mean with standard deviation. The *p* value reports the significance of the differences tested by the Kruskal–Wallis test for continuous variables and the X^2 test for categorical variables. ALD, alcohol-related liver disease; ALT, alanine transaminase; AST, aspartate aminotransferase; Cap, controlled attenuation parameter; CPA, C-reactive protein; GGT, gamma-glutamyl transferase; HCs, healthy controls; MELD, model for end-stage liver disease; T2D, type 2 diabetes; TE, transient elastography.

hereafter. The Qiagen All Prep Power Fecal DNA/RNA Kit with an additional phenol-chloroform step after cell lysis was used for DNA extraction. Ribosomal RNA (rRNA) was depleted from the extracted RNA using the NEBNext Bacteria rRNA Depletion Kit (New England Biolabs, Ipswich, MA, USA). Pooled Library preparation was done using NEBNext Ultra II DNA library kit (metagenomics) and NEBNext Ultra II Directional RNA Library Prep Kit (metatranscriptomics; New England Biolabs) and dual index multiplex oligos while aiming for a 350–400 bp insert size. Sequencing of 2 × 150 bp paired-end reads was performed on an Illumina HiSeq 4000 platform (Illumina, San Diego, CA, USA). For metagenomics and metatranscriptomic sequencing, we used ngless[30] (v1.1) to filtered out low-quality and host reads, keeping only bases with Phred >25 and reads >45 nt. Reads representing human DNA were identified by comparing all read sequences to the human reference genome and any reads with >90% similarity were discarded. The median sequencing depth was 5.71 Gb per sample for metagenomes and 1.17 Gb per sample for metatranscriptomes.

For microbial taxonomic profiles, the metagenomic operational taxonomic units (mOTUs)[31] v2.5 tool was used together with Genome Taxonomy Database (GTDB)-tk[32] (v2.11 r207) to acquire GTDB taxonomy for the ref-mOTUs via proGenomes2[33] genomes. The meta-mOTUs taxonomy was determined using a rule-based system and an 80% consistency threshold within clusters against the GTDB marker genes.

For in-depth gene-ontology information, the sequencing reads were aligned to the sub-catalog of the human gut microbiome within the Global Microbial Gene Catalog[34] using BWA-MEM[35] (v0.7.17). We used the eggNOG-mapper[36] (v1.0.3) with the eggNOG database 5.0 to assign KEGG orthologies (knockout [KO]) to each gene. Read counts for each KO were counted using gffquant (v2.9.1);[37] while multiple mapping counts were split among the genes evenly. In parallel, the microbial functional pathways were estimated by HumanN3 with a joint-index strategy.[38,39] We constructed MAGs as described before[40] and taxonomically classified them using GTDB-tk[32] (v2.11 r207).

Data analysis

Levels of metabolites in plasma were tested against the liver fibrosis stage using the R-package *limma* (R Foundation for Statistical Computing, Vienna, Austria) for an analysis of covariance (ANCOVA) and *post-hoc* analysis, where metabolite level was explained by the fibrosis stage category. Metabolites, with any difference between the fibrosis stages, were tested further with pairwise comparisons between fibrosis stages. These metabolite patterns were validated in two external cohorts, where the agreement in effect sign was tested between the primary cohort and the two validation cohorts, each, with a binomial test. The estimated standardized effect sizes and metabolite levels concerning the fibrosis stage were visualized in a heatmap and boxplots, respectively. Finally, the independence of the associations was tested with covariate adjustment to age, sex, BMI, current abstinence from alcohol, glycated hemoglobin (HbA1c), homeostatic model for insulin resistance (HOMA-IR), systolic blood pressure and current statin medication. The same analysis strategy was repeated for metabolite levels in feces, as well as microbial taxa and microbial KEGG orthologs in feces. For the linear models, the microbial derived profiles were log10 normalized.

Spearman cross-correlation was calculated between omics within the main dataset. We filtered liver proteins, and microbial genes focusing on bile acid pathways and applied abundance (1e-4 relative abundance) and prevalence (at least measured in 10% of the samples) filter on the microbial species. We only performed correlations between different omics and not between microbial species and microbial genes abundances as both are derived from the same omic (metagenomics). We corrected for multiple testing using Benjaminig-Hochberg fdr correction.

Differential abundance analysis between two groups of patients was performed to investigate differences in the abundance of microbial pathways (abundance filter = 1e-7) in the fecal samples and proteins in the liver tissue (abundance filter = 1e-05). In the linear models and differential abundance analysis, the *p* values were corrected for multiple testing using the Benjamini–Hochberg correction.[41]

Results

Levels of circulating and fecal bile acids increase during ALD progression

Targeted metabolomics of circulating metabolites revealed that fibrosis associated with elevated plasma levels of the conjugated primary BAs taurocholic acid (TCA, Fig. 1A), glycocholic acid (GCA, Fig. 1B), the primary BA cholic acid (CA, Fig. 1E), and the conjugated secondary BAs glycoursodeoxycholic acid (GUDCA, Fig. 1C) and tauroursodeoxycholic acid (TUDCA, Fig. 1D). Taurine, a substrate for BA conjugation, showed decreased plasma levels with advancing fibrosis (Fig. 1F). All six metabolites remained independently associated with fibrosis upon adjustment for clinical covariates. These trends were confirmed in both validation cohorts when comparing advanced fibrosis against minor-significant fibrosis (Fig. 1F, binomial test for effect signs; p <0.05), whereas the pattern in individual fibrosis stages was inconclusive because of small sample size (Fig. S1). Other measured metabolites did not show statistically significant changes (Fig. S2).

In fecal samples, the primary BA chenodeoxycholic acid (CDCA, Fig. 2C) and its taurine-conjugated form taurochenodeoxycholic acid (TCDCA, Fig. 2F) increased in abundance up to fibrosis stage three but declined at stage four. Similarly, CA (Fig. 2A) and its gut microbiome-produced derivative deoxycholic acid (DCA, Fig. 2E) showed increased levels with advancing fibrosis. Additionally, azelaic acid decreased with fibrosis progression (Fig. 2B). Upon adjustment to clinical covariates, azelaic acid, CA, CDCA, and TCDCA remained independently associated with fibrosis. Although the trends for CDCA and DCA were consistent across the main and both validation cohorts, CA and TCDCA levels displayed heterogeneity across the validation cohorts (Fig. 2F and Fig. S3). Other measured metabolites did not show statistically significant changes (Fig. S4).

Dysregulation of human bile acid metabolism in ALD

The abnormal levels of primary and secondary conjugated BAs found in the plasma and feces of patients with ALD indicate altered BA metabolism in the liver and impaired hepatic extraction. We analyzed 4,765 liver proteins measured by mass spectrometry and identified 1,146 proteins that were significantly different between low (F0–1) and high fibrosis (F2–4) groups (Fig. S5). Four enzymes involved in primary BA biosynthesis were progressively downregulated with increasing liver fibrosis: CYP8B1, AKR1D1, BA-CoA-ligase (BAL, S27A5), and AKR1C4 (Fig. 3). CYP8B1 (cytochrome P450 family 8 subfamily B member 1) encodes the enzyme 7-α-hydroxycholest-4-en-3-one 12-α-hydroxylase, a key protein responsible for synthesizing CA from cholesterol.[42] AKR1D1 encodes aldo-keto reductase family 1 member D1, which is essential for the production of cholic acid.[43]

Genes encoding solute carrier organic anion transporters of family 1B members (SLCO1B1 and SLCO1B3), which mediate hepatic uptake of endogenous compounds such as BAs and bilirubin,[44] were downregulated with advancing fibrosis (Fig. 3). Export mechanisms protecting hepatocytes from cytotoxic BA accumulation were impaired as well. Expression of the bile salt export pump (BSEP), encoded by ABCB11, decreased with fibrosis progression. BSEP is the main transporter of hydrophobic BAs across the canalicular membrane of hepatocytes.

The phosphatidylcholine translocator (encoded by ABCB4) was significantly upregulated with increasing fibrosis. This protein facilitates the excretion of phospholipids from the liver into bile, where they form mixed BA–phospholipid micelles, protecting biliary epithelial cells from irritation caused by high concentrations of bile salts.[43] The hepatic SLCO1B3 protein level had moderate inverse correlation with levels of plasma BAs GCA, GUDCA, and TCA (Fig. S6 and Table S2).

Microbial bile acid epimerization and dehydroxylation pathway abundances increase in ALD

Microbes in the human intestine play a major role in BA homeostasis, as they metabolize the BA to their secondary forms and are increasingly recognized as important modulators of liver diseases.[5] We investigated microbial functional profiles regarding their secondary BA metabolism. Among 549 microbial pathways detected in the main cohort, the BA epimerization and the 7-β-dehydroxylation pathway were weak but differentially enriched in individuals with ALD compared with the healthy controls (Fig. S7A and Table S6). Within the ALD cohort, comparing low fibrosis (F0–1) to high fibrosis (F2–4) groups, BA epimerization pathways increased in abundance with more advanced fibrosis (Fig. S7B and Table S7).

We further analyzed the abundance of secondary BA pathway gene orthologs in metagenomic data (metaG) and their expression in metatranscriptomic data (metaT). The multistep dehydroxylation of the primary BAs CA and CDCA to the secondary BAs DCA and lithocholic acid (LCA) by gut microbes is encoded by the BA-inducible (*bai*) genes.[19] We found individual *bai* operon genes to be significantly altered in their abundance (Fig. 4A–C). Genes encoding early oxidation steps, *baiA* (Fig. 4G)*, baiH* (Fig. 4E)*, baiF* (Fig. 4F)*,* and *baiB* (Fig. 4D), were enriched in ALD.

Although statistical significance in the metatranscriptomic data was less robust than in metagenomic data, partly because of the lower sequencing depth and mapping rates, similar trends were generally observed (Fig. 4B and D–H). *BaiN*, a gene encoding the reduction arm of the dehydroxylation pathway,[20] showed the highest overall abundance in the microbiomes, but was strongly depleted in both gene and transcript levels with ALD and advanced fibrosis (Fig. 4C and H). When considering the influence of clinical covariates, the metagenomic amounts of baiF, baiH, and baiN remained independently associated with ALD, and the metagenomic depletion of baiN remained independently associated with fibrosis. In the metatranscriptome, baiA, baiN, and hdhA remained independently associated with ALD after adjustment for clinical covariates.

Enzymes encoded by 7-α-hydroxysteroid dehydrogenase (*hdhA*, also known as 7-α-*hsdh*) contribute to secondary BA diversity by promoting oxidation and partly epimerization, producing derivatives like ursocholic acid (UCA) and

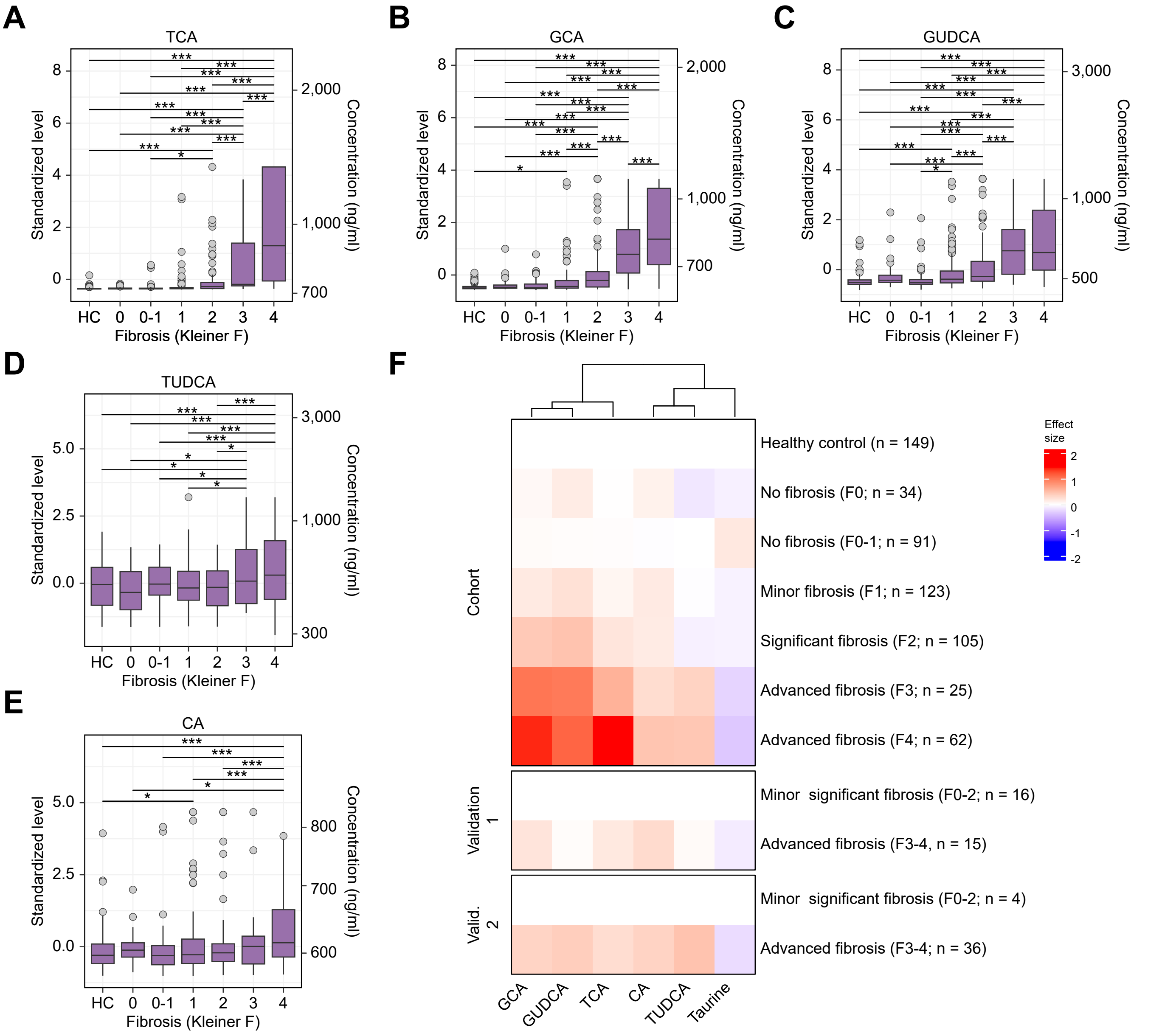

Fig. 1. Serum metabolite levels according to fibrosis stage. (A–E) Boxplot of metabolite level (y-axis: standardized level, left; concentration, right) grouped by fibrosis stage (x-axis) in the main cohort and the healthy control (HC) group. Pairwise differences are highlighted as ***multiple testing-corrected p <0.05; *nominal p <0.05. (F) Heatmap of the standardized effect size of metabolite level as compared with healthy controls (main cohort) or ALD with no or minor fibrosis (validation cohorts). Differentially abundant metabolites are shown in columns (ordered by hierarchical clustering), different cohorts and fibrosis stages in rows, and the respective effect size in color (red: increase; blue: decrease). ALD, alcohol-related liver disease; CA, cholic acid; GCA, glycocholic acid; GUDCA, glycoursodeoxycholic acid; TCA, taurocholic acid; TUDCA, tauroursodeoxycholic acid.

ursodeoxycholic acid (UDCA), and their taurine and glycine-conjugated forms.[16] This pathway is favorable for both microbiota and host, as it detoxifies the BA pool by converting hydrophobic BAs into more hydrophilic forms.[45],[46]

Identification of bacterial species most responsible for altered BA metabolism

To pinpoint the microbial species driving the metabolic changes during ALD progression, we mapped KEGG orthologous genes from our dataset onto MAGs constructed from samples from the same main cohort of individuals with ALD and HCs. MAG assembly yielded 32,336 bins, of which 3,746 high-quality (>95% completeness, <5% contamination) and 15,914 medium-quality (>50% completeness, <10% contamination) bins were used for downstream analysis. This approach allowed us not only to assess species abundance fold changes, but also to calculate the fraction of MAGs from each species carrying the genes of interest. We successfully mapped the *bai* genes with the highest overall abundance (*baiN*, K07007), the highest fold change (*baiB*, K15868), and the gene responsible for BA oxidation (*hdhA*, K00076; Fig. 4). Further, we mapped the metatranscriptomic reads assigned to the KEGG orthologous onto the MAGs to estimate the

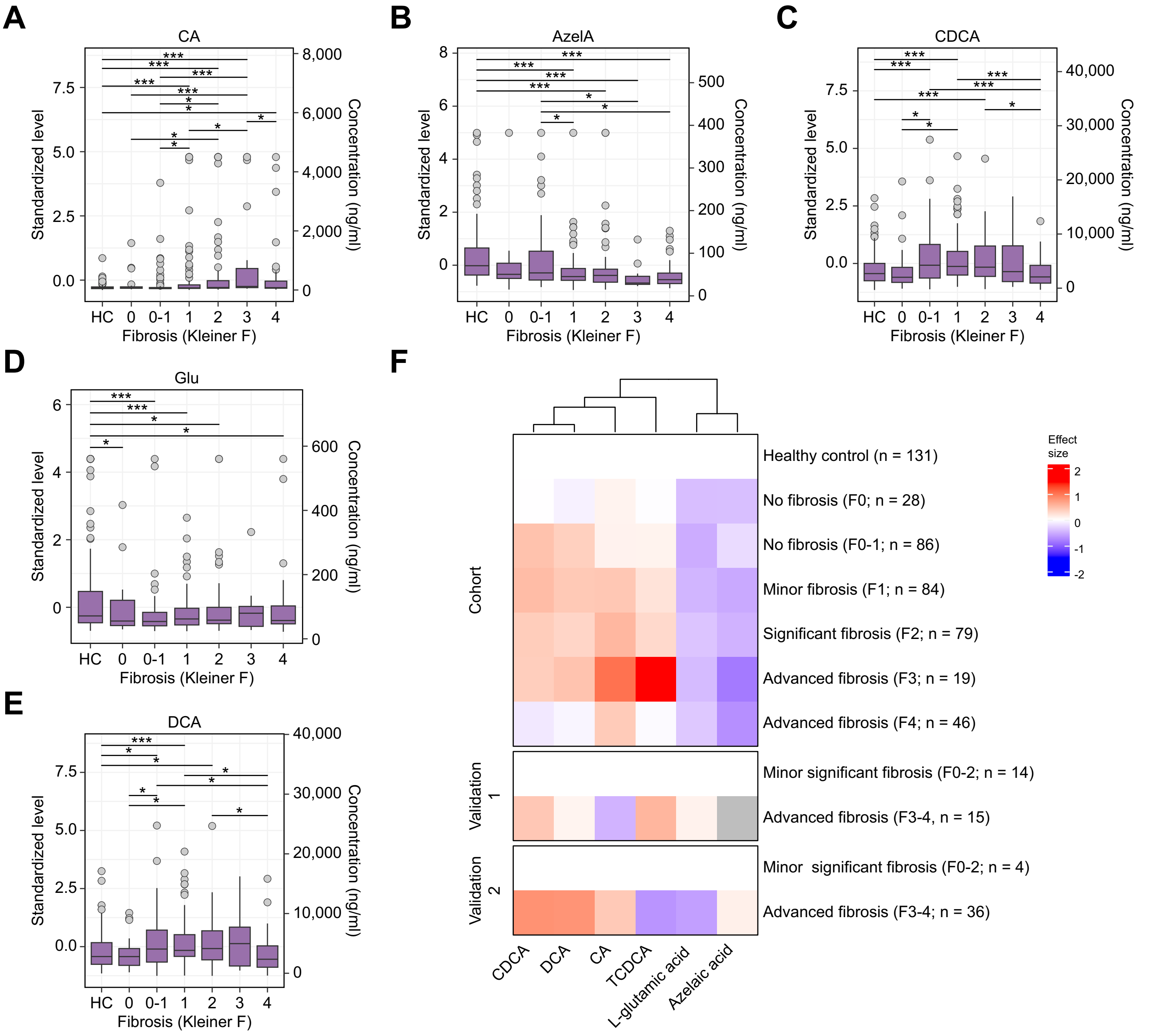

Fig. 2. Fecal metabolite levels according to fibrosis stage. (A–E) Boxplot of metabolite level (y-axis: standardized level, left; concentration, right) grouped by fibrosis stage (x-axis) in the main cohort and the healthy control (HC) group. Pairwise differences are highlighted as ***multiple testing-corrected p <0.05; *nominal p <0.05. (F) Heatmap of the standardized effect size of metabolite level as compared with healthy control (main cohort) or ALD with no or minor fibrosis (validation cohorts). Differentially abundant metabolites are shown in columns (ordered by hierarchical clustering), cohorts and fibrosis stages in rows and the respective effect size in color (red: increase; blue: decrease; gray: not available). ALD, alcohol-related liver disease; CA, cholic acid; CDCA, chenodeoxycholic acid; DCA, deoxycholic acid.

transcription of the genes by the bacteria (Fig. S8). In addition, we investigated the bile salt hydrolase gene (*bsh*; in KEGG choloylglycine hydrolase = *cbh*, K01442), as it encodes a key 'gatekeeper' reaction in secondary BA metabolism. The deconjugation of glycine and taurine from primary BAs is a crucial initial step enabling subsequent BA transformations.[47]

The *bsh* gene is widely distributed across the bacterial tree of life,[48] reflected here by 753 species identified as carrying *bsh* within our study population (Fig. 5A). Although the overall abundance of *bsh* did not change (Fig. 4C), we identified *Blautia_A wexlerae* and *Parabacteroides distasonis* as key species with increased abundance during disease progression with a high fraction of gene-carrying MAGs (Fig. S9) and active expressions (Fig. S8). We identified positive correlations between the *bsh* gene abundance and the abundance of the deconjugated BA CDCA (ρ = 0.18), and DCA (ρ = 0.16) in the main dataset (Fig. S10).

Eggerthella lenta may facilitate the oxidation arm of the BA dehydroxylation pathway, whereas the BaiN gene for the reduction arm is prevalent across many taxa

The CoA ligase *baiB* gene, encoding the first enzyme of the oxidation arm of the dehydroxylation pathway, was originally

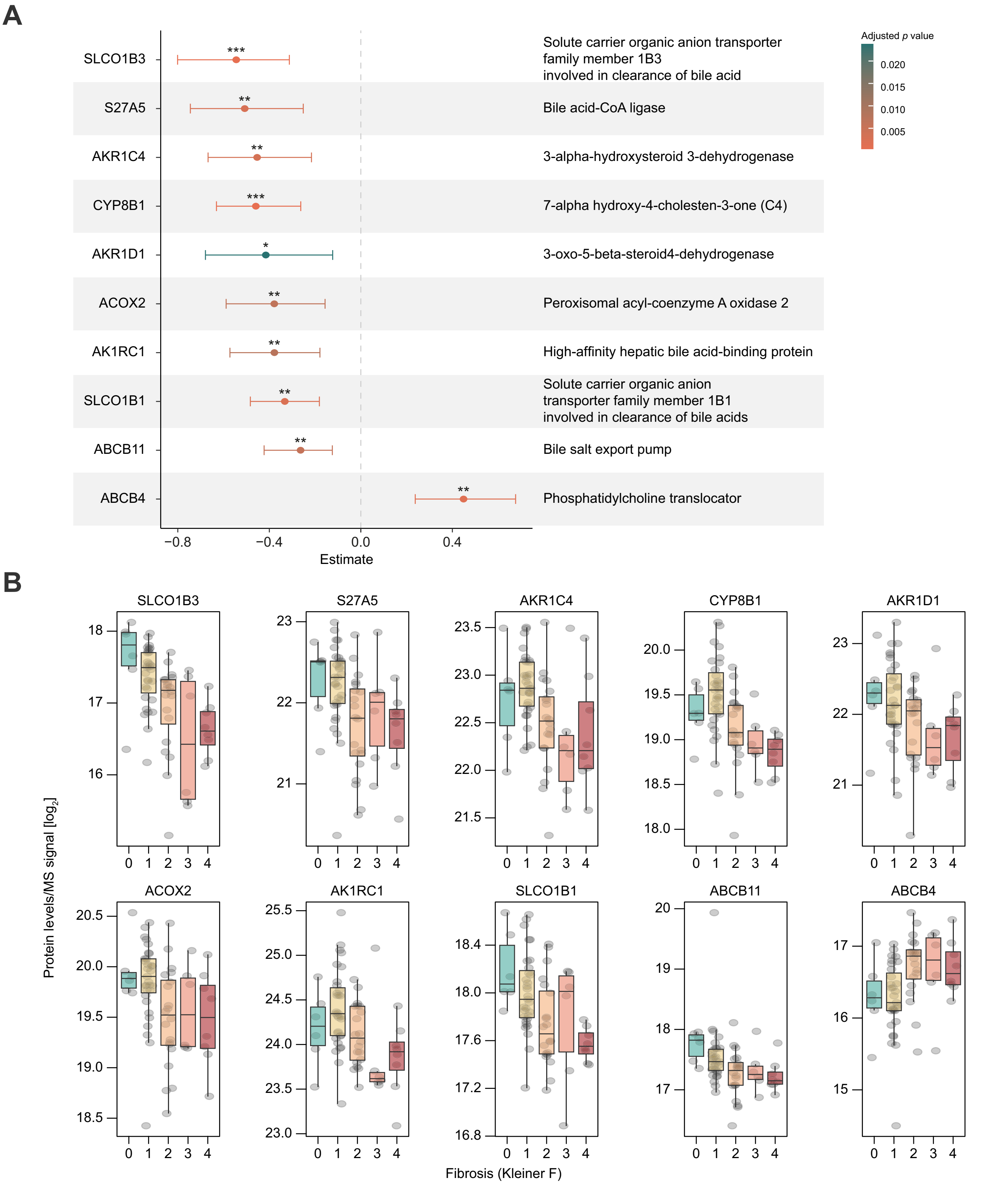

Fig. 3. Liver protein changes their level upon fibrosis. (A) BA metabolism and transport related liver proteins that significantly change between patients with no or minor fibrosis (F0–1; n = 37) and patients with moderate or advanced fibrosis (F2–4; n = 34) within the ALD cohort. We performed Wilcoxon rank-sum tests and subsequent Benjamini–Hochberg correction for false discovery rate on the whole dataset with 4,765 liver proteins. Shown is the estimated median shift between groups (F2–4 - F0–1) with 95% CIs. Negative estimates correspond to a decrease in abundance upon fibrosis progression. *q <0.05, **q <0.01, ***q <0.001. (B) Protein expression of the liver proteins from panel A is shown according to fibrosis stage. BA, bile acid.

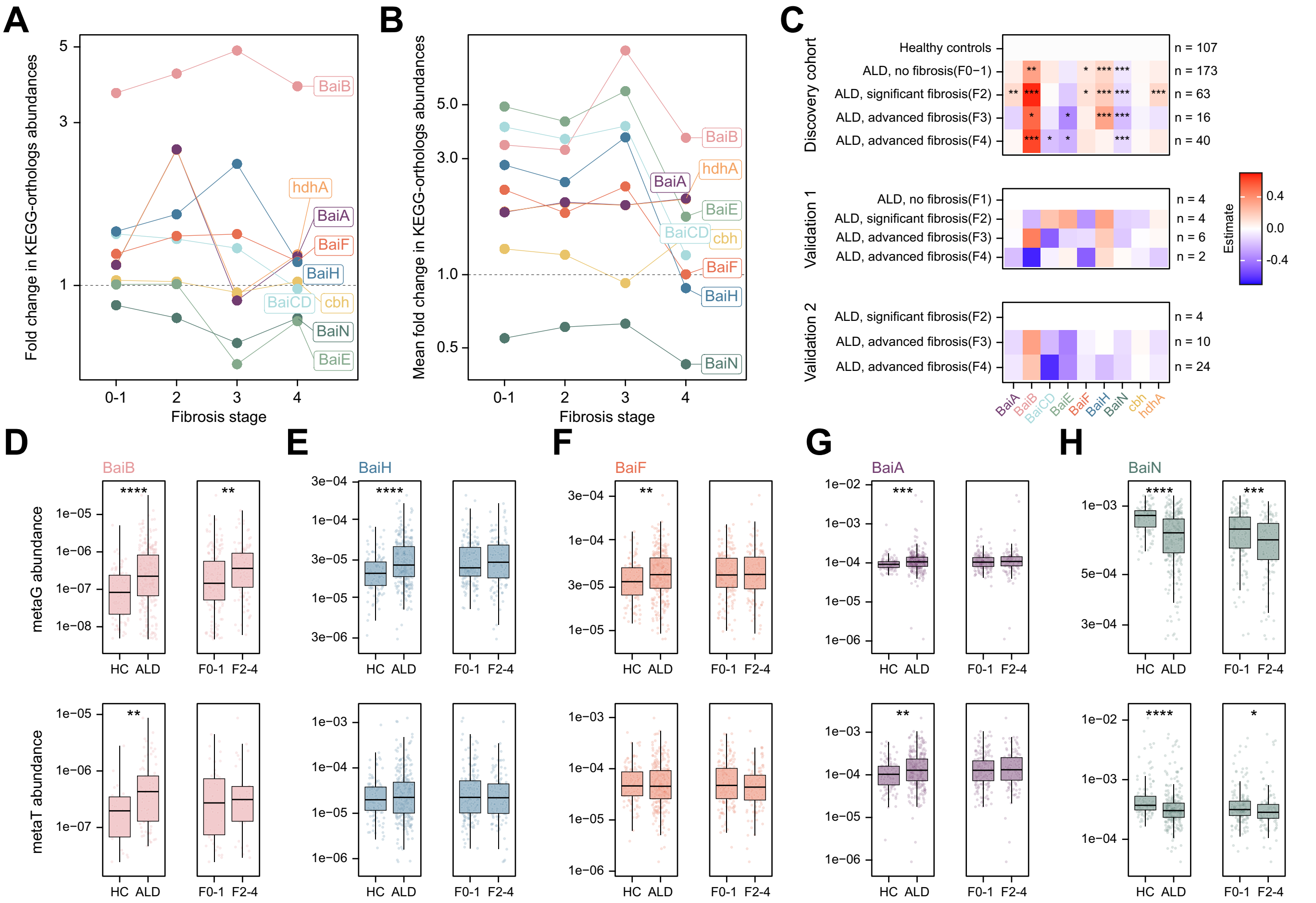

Fig. 4. Microbial genes of the secondary BA metabolism change upon fibrosis stages. (A, B) Abundance fold change of microbial secondary BA metabolism KEGG orthologs genes derived from metagenomic (metaG) sequencing (A) and gene expression derived from metatranscriptomic (metaT) sequencing (B) for each fibrosis stage in comparison with the healthy control samples. (C) Heatmap of the linear model estimate of the abundance change for KEGG ortholog genes (log_{10} normalized) derived from metagenomic sequencing. Significance is reported after correcting for multiple testing using the Benjamini–Hochberg correction. *q <0.05, **q <0.01, ***q <0.001. (D–H) Comparison of KEGG ortholog gene abundance (metaG) and gene expression (metaT) between healthy controls (HC) and samples from ALD and within the ALD group between no or minor fibrosis (F0–1) and moderate or advanced fibrosis (F2–4). ALD, alcohol-related liver disease; bai, bile acid-induced; KEGG, Kyoto Encyclopedia of Genes and Genomes.

identified in *Clostridium hylemonea* and *Clostridium scindens*,[49] but was not mapped to these taxa in our cohort. The BA conversion ability of the *Eggerthella lenta* has previously been demonstrated *in vitro,* primarily focusing on the 3-, 4-, and 12-α-HSDH genes.[20] *Eggerthella lenta* has previously been reported to carry *bai* genes[50] but was considered to play only a minor role in secondary BA metabolism *in vivo.*[51] However, in our study population, *Eggerthella lenta* was highly enriched in patients with ALD and showed a high prevalence of MAGs containing the *baiB* gene (Fig. 5B). Metatranscriptomic reads, assigned to the BaiB genes, were mapped to the *Eggerthella lenta* MAGs indicating active transcription (Fig. S8). We also observed weak but nominally significant positive correlation between *Eggerthella lenta* abundance and fecal and plasma levels of LCA ($\rho_{feces} = 0.1$, $\rho_{plasma} = 0.13$) and DCA ($\rho_{feces} = 0.1$, $\rho_{plasma} = 0.15$), the products of BA dehydroxylation (Fig. S11). However, further investigations are needed to validate the BA dehydroxylation potential of *Eggerthella lenta*.

We identified 1,037 species as carriers of the 3-dehydro-bile acid Δ4,6-reductase *baiN* gene (Fig. 5A). The *baiN* gene was experimentally characterized and identified in *Clostridium scindens*,[52,53] and homologs have been detected across the families *Ruminococcaceae*, *Lachnospiraceae*, and *Peptostreptococcaceae.*[54] *Fusocatenibacter saccharivorans*, a member of *Lachnospiraceae,* showed a high prevalence in MAGs containing *baiN* but was depleted in patients with ALD in our analysis and previous reports on liver cirrhosis.[55]

Mediterraneibacter torques and *Bacteroides thetaiotaomicron* are presumably drivers of BA oxidation in ALD

MAGs containing the BA oxidation pathway-associated 7-α-*hsdh* KEGG ortholog (*hdhA*, *hsdh*, K00076) were identified in 197 species, many of which also possess other key genes (Fig. 5A). Numerous species carrying *hdhA* are enriched in ALD, with *Mediterraneibacter torques* and *Bacteroides*

thetaiotaomicron showing a near-universal presence and transcription of *hdhA* across their MAGs and notable prevalence in ALD samples, underscoring their role in BA metabolism in patients with ALD (Fig. 5D and Fig. S8). Previously, *hdhA* activity has been observed in *Clostridium*, *Eubacterium,* and *Ruminococcus* species.[56,57] In our cohort, we found *hdhA* in several *Clostridium* species and *Blautia_A wexlerae.* Indeed, the abundance of *Blautia_A wexlerae* was positively correlated with the fecal levels of the unconjugated primary BAs CA and CDCA (Table S5 and Fig. S11). Although *hdhA* is ubiquitously present in various *Clostridium* species, its low prevalence in the cohort suggests limited relevance to BA metabolism in ALD.

Integrative analysis of bile acid-related features between the omics

Finally, we investigated how BA levels correlated with the abundance of hepatic proteins, microbial genes and transcripts from the BA pathways as well as the abundance of microbial species. The levels of plasma BAs, primarily GUDCA, GCA, and TCA, correlated inversely with the hepatic levels of proteins in the BA-related pathways, including AKR1C4, SLC27A5 and SLCO1B3 (Fig. S6 and Table S2). Correlations between fecal BAs and hepatic proteins were fewer and weaker, lead by GDCA with CYP39A1.

Microbial abundance of the gene *baiN* (K07007) was inversely correlated with BA levels both in feces and in plasma (Table S3 and Fig. S10). *BaiB*, *baiCD*, and *baiH* (K15868, K15870, and K15873, respectively) were positively correlated with fecal BAs, with the exception that *BaiCD* was inversely correlated with CA. *BaiN* and *aiB*, both, were also correlated with GCA and TCA in plasma. The microbial expression of the genes *baiA* (K15869), *baiCD*, *baiF* (K15871), *baiH* and *hdhA* (K00076) was positively correlated with the fecal level of CA (Table S4). Moreover, *baiH* was correlated with DCA in plasma.

Correlations between microbial abundance and BA levels were numerous and lead by positive correlations with *Erysipelatoclostridium ramosum*, *Enterocloster bolteae*, and *Ruminococcus_B gnavus* (Table S5). The abundance of *Eggerthella lenta* was positively correlated with plasma levels of GCA, GUDCA, and TCA. Moreover, in feces it was positively correlated with CA and inversely correlated with TUDCA. *Blautia_A wexlerae* was positively correlated with fecal CA, CDCA, and DCA, and plasma GCA, GUDCA, and TCA. *Mediterraneibacter torques* was correlated with fecal DCA, GDCA, LCA, and TUDCA, and plasma GCA, TCA, and GUDCA.

Discussion

In this study, we observed increased BA abundance in the plasma of patients with ALD, accompanied by downregulation of proteins involved in BA production and transport. Elevated BA concentrations were also evident in fecal samples, reflecting excessive BA excretion. By mapping KEGG orthologous genes, we pinpointed the microbial taxa most responsible for changes in secondary BA metabolism, highlighting new targets for mechanistic studies.

Fig. 6 presents an integrated view of host and microbial BA metabolism in ALD. Primary BA production of CA and CDCA from cholesterol is reduced in the liver, as demonstrated by decreased levels of biosynthetic enzymes in the classical pathway. Suppression of CYP8B1 has been shown to reduce CA synthesis and to increase the hydrophobicity of the BA pool via accumulation of 7α-hydroxy-4-cholesten-3-one (7αC4),[58] an extremely hydrophobic and cytotoxic BA reported to cause liver cell damage.[59] Deficiency in AKR1D1 leads to accumulation of toxic intermediates such as 3-oxo-Δ4 BAs and allo-BAs in the liver, which is associated with progressive intrahepatic cholestasis, hepatocyte injury, and apoptosis.[60] To mitigate the excess of hepatotoxic BAs, compensatory mechanisms reduce BA accumulation in hepatocytes.[4] For example, primary BA synthesis is regulated by the nuclear farnesoid X receptor (FXR) in the gut epithelium via a negative feedback mechanism.[14] Gut microbes can inhibit BA production by producing FXR antagonists that regulate CYP8B1 expression, the rate-limiting enzyme in BA synthesis.[15] Previously, GUDCA and TUDCA have been identified as FXR antagonists[61] and are both upregulated with fibrosis progression in our cohort. As FXR is further involved in various pathways associated with hepatocyte protection and antifibrotic effects,[62] its inhibition by elevated levels of GUDCA and TUDCA likely further drives the fibrosis progression. Excessive alcohol consumption in ALD disrupts the enzymatic detoxification, alters microbial composition,[63] and compromises intestinal barrier function.[64,65] This interplay between disrupted liver BA metabolism and gut microbial alterations, combined with impaired FXR signaling, likely exacerbates leaky gut and microbial translocation, creating a vicious cycle of liver injury and inflammation.

Our study shows dysregulation of several enzymes that are crucial for the BA transport across different compartments in ALD (Fig. 6C). Downregulation of both SLCO1B1 and SLCO1B3, responsible for the hepatic uptake of BAs from blood, has been previously associated with Rotor syndrome, a condition characterized by high concentrations of plasma unconjugated BAs and bilirubin. These transporters compensate for the dysfunction of the main uptake transporter, the NA+-dependent taurocholate cotransporting polypeptide (NTCP).[66] NTCP is recognized as the main uptake transporter for conjugated BAs from plasma to the liver, and several studies have demonstrated that its deficiency is related to highly elevated concentrations of conjugated BAs in the blood.[66–68] In our study, NTCP was undetectable in more than 70% of the liver samples analyzed via MS-proteomics, as the levels were below the detection/quantification limit, suggesting that this transporter might be downregulated in ALD as it has been previously demonstrated in cholestatic diseases.[69,70] It is important to highlight that the undetectable levels of NTCP might result from technical limitations in the sensitivity of the MS-proteomics methodology implemented in the analysis. The simultaneous downregulation of SLCO1B1, and SLCO1B3 indicates impaired hepatic BA extraction, explaining the increased plasma concentrations of TCA, GCA, TUDCA, and GUDCA observed with fibrosis progression. Especially TCA and GCA play an important role in the progression of fibrosis, as they activate the sphingosine-1-phosphate receptor 2 (S1PR2) expression in hepatic stellate cells,[62] the main drivers of fibrogenesis. Conjugated BAs such as TCA also induce the activation of S1PR2 expression in macrophages, inducing the production of proinflammatory cytokines that initiate a signaling cascade that links chronic inflammation to fibrogenesis.[71] In mouse models, this activation by TCA and GCA was associated with the MAPK/YAP pathway, promoting cell

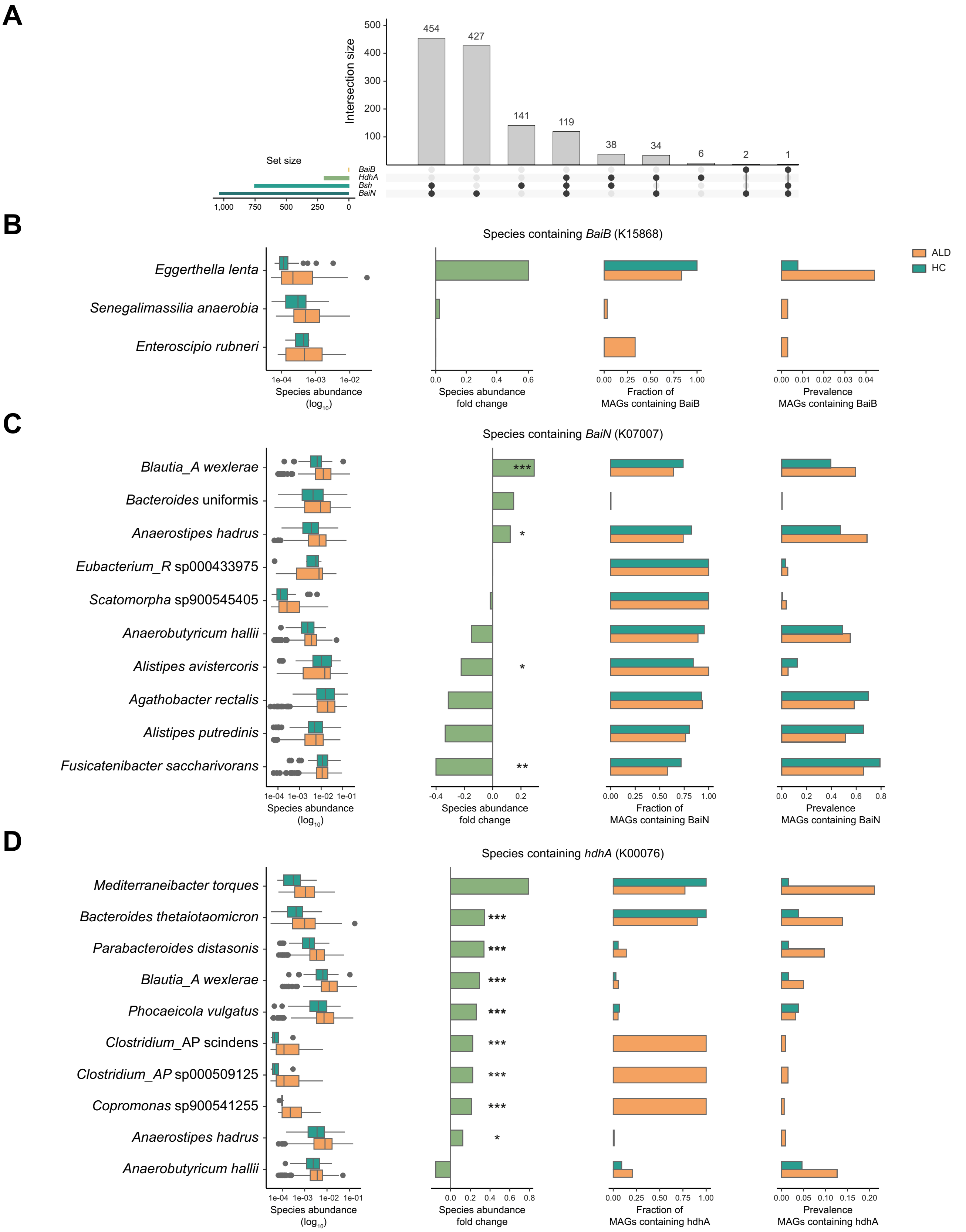

Fig. 5. Importance of microbial taxa for secondary BA metabolism. (A) Intersection of bacterial species containing the four genes of interest. (B) Microbial taxa responsible for the secondary BA metabolism dehydroxylation pathway are facilitated by the *bai* operon. For *BaiB*, the gene was only found on MAGs from three different taxa. (C) Microbial taxa responsible for the reduction arm of the dehydroxylation pathway. For the *BaiN* gene, the 10 species with the highest fold change abundance between ALD and HC are shown. (D) Microbial taxa for secondary BA metabolism oxidation facilitated by *hdhA.* Importance is collectively estimated by

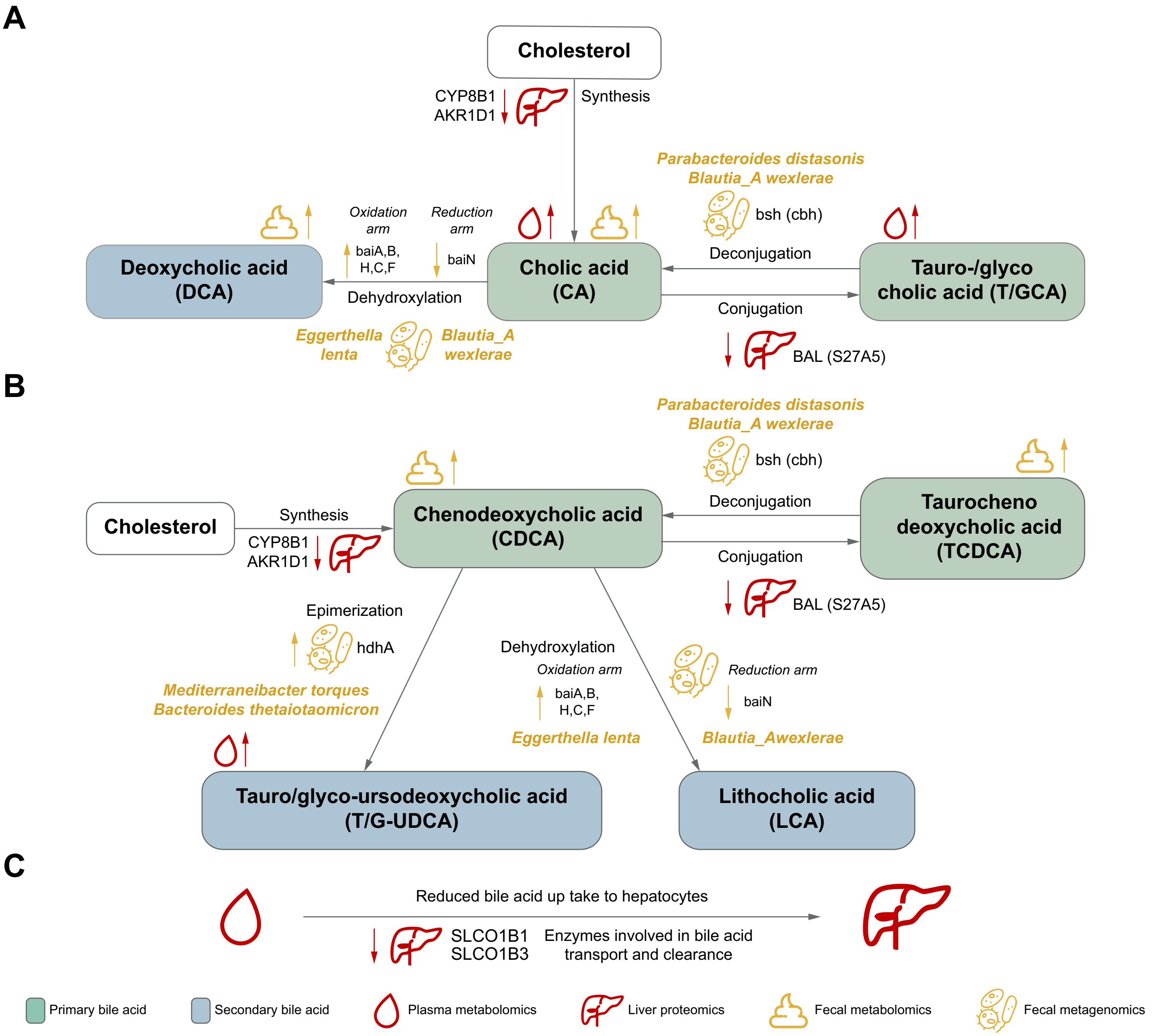

Fig. 6. Combined representation of bile acid synthesis changes upon alcohol-related liver disease. (A, B) The primary BAs cholic acid (A) and chenodeoxycholic acid (B) are metabolized by secondary BA metabolism. (C) Primary BA metabolism and transport by liver proteins. (A–C) *bai*, bai operon; *Bsh*, bile salt hydrolase (also *cbh* = choloylglycine hydrolase), *hdhA*, 7-alpha-hydroxysteroid dehydrogenase.

growth and proliferation, resulting in increased fibrosis and inflammation.[72]

Loss of the BSEP contributes to hepatocellular accumulation of toxic BAs, leading to intrahepatic cholestasis.[73] However, the phosphatidylcholine transporter ABCB4 was significantly upregulated with increasing liver fibrosis. ABCB4 facilitates the excretion of phospholipids into bile, forming mixed BA–phospholipid micelles that protect biliary epithelial cells from irritation caused by high BA salt concentrations. The homeostasis of this protective synergy between BSEP and ABCB4 appears to be disrupted in alcohol-related fibrosis.[74]

Alcohol overuse was shown to induce translocation of microbes and their metabolites, including BAs, because of compromised intestinal barrier function.[64,65] The most cytotoxic secondary BAs are LCA and DCA; the latter was found enriched in the feces of patients with ALD, possibly

the species abundance, species fold change between healthy and ALD samples, the gene-carrying fraction of MAGs. The prevalence of gene-carrying MAGs in the cohort indicates the fraction of samples containing the MAGs in our cohort. Significance levels in the fold change are derived from a differential abundance analysis (Wilcoxon rank-sum test) and corrected for multiple testing: *p ≤0.05, **p ≤0.01, ***p ≤0.001. ALD, alcohol-related liver disease; BA, bile acid; bai, bile acid-induced; HC, healthy control; hdhA, 7-alpha-hydroxysteroid dehydrogenase; MAG, metagenome-assembled genome.

contributing further to intestinal permeability.[75] These BAs have been implicated in cell membrane disruption, bile duct damage, inflammation, and cholestasis, causing hepatocyte injury, liver fibrosis, and liver cancer.[76–78] The *bai* operon governs dehydroxylation of primary BAs to LCA and DCA in the gut, and is well described, for example, in *Clostridium* species.[53] However, *Clostridium* species were not a dominant carrier of *bai* genes in our cohort. Instead, *Eggerthella lenta* and *Blautia_A wexlerae* seem to assume key roles in BA dehydroxylation in ALD but lack the full enzymatic pathway. *Eggerthella lenta* has been reported to harbor *bai*-similar genes with yet unknown function.[20] As also the abundance of *Eggerthella lenta* was increased in patients with ALD, it emerges as an interesting microbe with different and yet to determine BA metabolic capacities. We found early *bai* genes to be enriched, whereas the late-pathway gene *baiN*, although highly abundant overall, strongly decreased with disease progression, indicating a metabolic bottleneck. This community-level dysfunction likely results from microbial dysbiosis induced by alcohol overuse. We hypothesize that impaired conversion to secondary BAs and accumulation of toxic intermediates may exacerbate intestinal barrier disruption, fostering a vicious cycle of increased permeability, bacterial products, and BA translocation to the liver, and hepatic inflammation.[79]

The secondary BAs UDCA and TUDCA exhibit low toxicity and anti-inflammatory properties and are clinically used to treat cholestatic liver disease and biliary cirrhosis.[80,81] We observed increased TUDCA levels in blood and elevated abundance of the *hdhA* gene, responsible for UDCA and TUDCA production, peaking at fibrosis stage two. However, *hdhA* levels declined in cirrhosis and advanced fibrosis across cohorts, and TUDCA was further depleted in advanced fibrosis in validation cohort 1.

We also found that azelaic acid levels in feces decreased with fibrosis progression. Azelaic acid is known to have anti-inflammatory, antioxidant, and antibacterial properties. Further, azelaic acid has demonstrated anti-atherosclerotic action,[82] and protects the liver against oxidative stress induced by ethanol and high-fat diets.[83]

Our cross-sectional study design presents a limitation to inferring cause and effect in the findings, and thus, our findings are to be interpreted as associations. Moreover, the small sample size in the validation cohorts, as well as their non-uniform distribution of fibrosis levels, sets a limit on the power of validation. In the main cohort, BA levels across fibrosis stages were compared with levels from healthy controls, whereas in the validation cohorts, early and late fibrosis stages were compared. This difference in comparison approaches may account for some discrepancies, such as the observed reduction of TCDCA at stage four. Further, liver tissue proteomics was performed in bulk and normalized by total protein amount; the observed fibrosis-associated protein changes may reflect both alterations in cellular composition and cell-intrinsic regulation. In addition, the microbial functional analyses rely on gene-ontology databases, which often lack mechanistic proof for individual taxa and are limited to genes available in the databases.

The strengths of the present study, however, lie in the detailed characterization of the liver disease and deep phenotyping with various omics technologies. Unlike prior studies focusing on individual aspects of BA metabolism, we present a comprehensive integrated analysis of the whole gut–liver–BA axis and its disruption in ALD.

Affiliations

[1]*European Molecular Biology Laboratory, Molecular Systems Biology Unit, Heidelberg, Germany;* [2]*Steno Diabetes Center Copenhagen, Herlev, Denmark;* [3]*Fibrosis, Fatty Liver and Steatohepatitis Research Centre Odense (FLASH), Department of Gastroenterology and Hepatology, Odense University Hospital, Odense, Denmark;* [4]*Department of Clinical Research, Faculty of Health Sciences, University of Southern Denmark, Odense, Denmark;* [5]*IDEA, Global Research, Research and Development, Novo Nordisk, Copenhagen, Denmark;* [6]*Novo Nordisk Foundation Center for Protein Research, Faculty of Health and Medical Sciences, University of Copenhagen, Copenhagen, Denmark;* [7]*Novo Nordisk Foundation Center for Basic Metabolic Research, Faculty of Health and Medical Sciences, University of Copenhagen, Copenhagen, Denmark;* [8]*Department of Proteomics and Signal Transduction, Max Planck Institute of Biochemistry, Martinsried, Germany;* [9]*Max Delbrück Centre for Molecular Medicine, Berlin, Germany;* [10]*Department of Bioinformatics, Biocenter, University of Würzburg, Würzburg, Germany;* [11]*King's College London, London, UK*

Abbreviations

ALD, alcohol-related liver disease; ALT, alanine transaminase; AST, aspartate aminotransferase; BA, bile acid; *bai*, bile acid-induced; BAL, bile acid CoA ligase; BSEP, bile salt export pump; bsh, bile salt hydrolase; CA, cholic acid; CAP, controlled attenuation parameter; CDCA, chenodeoxycholic acid; CPA, C-reactive protein; DCA, deoxycholic acid; FXR, farnesoid X receptor; GCA, glycocholic acid; GGT, gamma-glutamyl transferase; GTDB, Genome Taxonomy Database; GUDCA, glycoursodeoxycholic acid; HC, healthy control; hdhA, 7-α-*hsdh*, 7-alpha-hydroxysteroid dehydrogenase; KEGG, Kyoto Encyclopedia of Genes and Genomes; KO, knockout; LCA, lithocholic acid; MAG, metagenome-assembled genome; MELD, model for end-stage liver disease; MetaG, metagenomics; MetaT, metatranscriptomics; mOTU, metagenomic operational taxonomic units; NTCP, NA+-dependent taurocholate cotransporting polypeptide; rRNA, ribosomal RNA; S1PR2, sphingosine-1-phosphate receptor 2; T2D, type 2 diabetes; TCA, taurocholic acid; TCDCA, taurochenodeoxycholic acid; TE, transient elastography; TUDCA, tauroursodeoxycholic acid; UCA, ursocholic acid; UDCA, ursodeoxycholic acid; VCTE, vibration-controlled transient elastography.

Financial support

This work was supported by funding from the European Union's Horizon 2020 research and innovation program under grant agreement number 668031 (GALAXY). This reflects only the authors' views, and the European Commission is not responsible for any use that may be made of the information it contains. The study was also supported by the Novo Nordisk Foundation through a Challenge Grant 'MicrobLiver' (grant number NNF15OC0016692). The work was supported by the EMBL International PhD Programme (MIK), by the Deutsche Forschungsgemeinschaft (DFG, German Research Foundation) - project number 460129525 (NFDI4Microbiota), and EMBL IT Services HPC resources.

Conflicts of interest

The authors declare no competing interests.
Please refer to the accompanying ICMJE disclosure forms for further details.

Authors' contributions

Conceptualization: MIK, AZ, MT, CLQ, PB, AK, TH. Data curation: AZ, TS, LN, AF, WA, CS, DP, CYK, AW, MK, MIK, HBJ, SN. Data analysis: AZ, TS, MIK, SN. Sample collection: MT, AK, JKH, MI. Sample processing AZ, DH, LN, MIK. Writing: AZ, MIK, CLQ, MK, DP. Visualization: MIK, AZ, TS, KS. Supervision: CLQ, KS, MK, PB. Funding acquisition: AK, TH, CLQ, PB. Discussed the results, reviewed the manuscript, and approved the final manuscript: all authors.

Data availability

Shotgun metagenomic data sequenced in the GALAXY/MicrobLiver consortia are publicly available in the European Nucleotide Archive under the accession numbers of BioProject: PRJEB76661 (GALA-ALD), BioProject: PRJEB76664 (GALA-HP), PRJEB76667 (Validation 1, GALA-RIF), and PRJEB76668 (Validation 2, GALA-POSTBIO). Clinical contextual data, proteomics, and targeted metabolomics data cannot be made publicly available owing to the higher need to maintain patient confidentiality. Averaged protein levels in the liver proteomes have been deposited in the GitHub repository (https://github.com/llniu/ALD-study, subfolder ALD-App). Permission to access and analyze data can be obtained following approval from the Danish Data Protection Agency and the ethics committee for the Region of Southern Denmark. The study protocol, standard operating procedures, and patient information are also available upon request.

GALAXY & MicrobLiver consortia

Torben Hansen, Matthias Mann, Jelle Matthijnssens, Aleksander Krag, Peer Bork, Manimozhiyan Arumugam, Jonel Trebicka, Morten Karsdal, Ema Anastasiadou, Hans Israelsen, Hans Olav Melberg, Cristina Legido-Quigley, Maja Thiele.

Clinical trials registration

GALAXY main cohort: Danish Data Protection Agency nos. 13/8204, 16/3492 and 18/22692; and Odense Patient Data Exploratory Network under study identification nos. OP_040 and OP_239. Validation cohort 1: EudraCT number 20214-001856-51. Validation cohort 2: ClinicalTrial.gov ID NCT03863730.

Acknowledgements

We thank all members of the GALAXY and MicrobLiver consortia for fruitful discussions and support. The authors are deeply grateful to Peer Bork for his invaluable guidance, vision, and contributions to this work. He passed away after the manuscript had been initially revised but before it was accepted. Finally, we express our deepest gratitude to all participants in the cohorts presented.

Supplementary data

Supplementary data to this article can be found online at https://doi.org/10.1016/j.jhepr.2026.101848.

References

Author names in bold designate shared co-first authorship

[1] **Yang Z**, **Kusumanchi P**, et al. Serum metabolomic profiling identifies key metabolic signatures associated with pathogenesis of alcoholic liver disease in humans. Hepatol Commun 2019;3:542–557.
[2] **Sanyal AJ**, **Ling L**, et al. Potent suppression of hydrophobic bile acids by aldafermin, an FGF19 analogue, across metabolic and cholestatic liver diseases. JHEP Rep 2021;3:100255.
[3] Manley S, Ding W. Role of farnesoid X receptor and bile acids in alcoholic liver disease. Acta Pharm Sin B 2015;5:158–167.
[4] Wei S, Ma X, Zhao Y. Mechanism of hydrophobic bile acid-induced hepatocyte injury and drug discovery. Front Pharmacol 2020;11:1084.
[5] Jiang L, Schnabl B. Gut microbiota in liver disease: what do we know and what do we not know? Physiology 2020;35:261–274.
[6] Sang C, et al. Bile acid profiles are distinct among patients with different etiologies of chronic liver disease. J Proteome Res 2021;20:2340–2351.
[7] Cremers CM, Knoefler D, Vitvitsky V, et al. Bile salts act as effective protein-unfolding agents and instigators of disulfide stress in vivo. Proc Natl Acad Sci U S A 2014;111:E1610–E1619.
[8] Kurdi P, Kawanishi K, Mizutani K, et al. Mechanism of growth inhibition by free bile acids in Lactobacilli and Bifidobacteria. J Bacteriol 2006;188:1979–1986.
[9] Dvorak K, Payne CM, Chavarria M, et al. Bile acids in combination with low pH induce oxidative stress and oxidative DNA damage: relevance to the pathogenesis of Barrett's oesophagus. Gut 2007;56:763–771.
[10] Gomaa EZ. Human gut microbiota/microbiome in health and diseases: a review. Antonie van Leeuwenhoek 2020;113:2019–2040.
[11] Wahlström A, Sayin SI, Marschall H-U, et al. Intestinal crosstalk between bile acids and microbiota and its impact on host metabolism. Cell Metab 2016;24:41–50.
[12] Galatola G, Jazrawi RP, Bridges C, et al. Direct measurement of first-pass ileal clearance of a bile acid in humans. Gastroenterology 1991;100:1100–1105.
[13] Chen P, Schnabl B. Host-microbiome interactions in alcoholic liver disease. Gut Liver 2014;8:237–241.
[14] Inagaki T, et al. Regulation of antibacterial defense in the small intestine by the nuclear bile acid receptor. Proc Natl Acad Sci U S A 2006;103:3920–3925.
[15] Sayin SI, et al. Gut microbiota regulates bile acid metabolism by reducing the levels of tauro-beta-muricholic acid, a naturally occurring FXR antagonist. Cell Metab 2013;17:225–235.
[16] Guzior DV, Quinn RA. Review: microbial transformations of human bile acids. Microbiome 2021;9:140.
[17] Azer SA, Hasanato R. Use of bile acids as potential markers of liver dysfunction in humans: a systematic review. Medicine 2021;100:e27464.
[18] Bhowmik S, Chiu H-P, Jones DH, et al. Structure and functional characterization of a bile acid 7α dehydratase BaiE in secondary bile acid synthesis. Proteins: Struct Funct Bioinformatics 2016;84:316–331.
[19] Funabashi M, Grove TL, Wang M, et al. A metabolic pathway for bile acid dehydroxylation by the gut microbiome. Nature 2020;582:566–570.
[20] Harris SC, DevendranS Mendéz-García C, et al. Bile acid oxidation by *Eggerthella lenta* strains C592 and DSM 2243T. Gut Microbes 2018;9:523–539.
[21] Downs DM. Understanding microbial metabolism. Ann Rev Microbiol 2006;60:533–559.
[22] Niu L, Thiele M, Geyer P, et al. Noninvasive proteomic biomarkers for alcohol-related liver disease. Nat Med 2022;28:1277–1287.
[23] Nishijima S, Stankevic E, Aasmets O, et al. Fecal microbial load is a major determinant of gut microbiome variation and a confounder for disease associations. Cell 2025;188:222–236.
[24] Rasmussen DN, Thiele M, Johansen S, et al. Prognostic performance of 7 biomarkers compared to liver biopsy in early alcohol-related liver disease. J Hepatol 2021;75:1017–1025.
[25] Israelsen M, Madsen BS, Torp N, et al. Rifaximin-α for liver fibrosis in patients with alcohol-related liver disease (GALA-RIF): a randomised, double-blind, placebo-controlled, phase 2 trial. Lancet Gastroenterol Hepatol 2023;8:523–532.
[26] Hansen JK, Israelsen M, Nishijima S, et al. The postbiotic ReFerm® versus standard nutritional support in advanced alcohol-related liver disease (GALA-POSTBIO): a randomized controlled phase 2 trial. Nat Commun 2025;16:5969.
[27] Kleiner DE, Brunt EM, Van Natta M, et al. Design and validation of a histological scoring system for nonalcoholic fatty liver disease. Hepatology 2005;41:1313–1321.
[28] de Zawadzki A, Thiele M, Suvitaival T, et al. High-throughput UHPLC-MS to screen metabolites in feces for gut metabolic health. Metabolites 2022;12:211.
[29] Ahonen L, Jäntti S, Suvitaival T, et al. Targeted clinical metabolite profiling platform for the stratification of diabetic patients. Metabolites 2019;9:184.
[30] Coelho LP, Alves R, Monteiro P, et al. NG-meta-profiler: fast processing of metagenomes using NGLess, a domain-specific language. Microbiome 2019;7:84.
[31] **Milanese A**, **Mende DR**, Paoli L, et al. Microbial abundance, activity and population genomic profiling with mOTUs2. Nat Commun 2019;10:1014.
[32] Chaumeil P-A, Mussig AJ, Hugenholtz P, et al. GTDB-Tk v2: memory friendly classification with the genome taxonomy database. Bioinformatics 2022;38:5315–5316.
[33] Mende DR, Letunic I, Maistrenko OM, et al. proGenomes2: an improved database for accurate and consistent habitat, taxonomic and functional annotations of prokaryotic genomes. Nucleic Acids Res 2020;48:D621–D625.
[34] Coelho LP, Alves R, Rodríguez Del Río Á, et al. Towards the biogeography of prokaryotic genes. Nature 2022;601:252–256.
[35] Li H, Durbin R. Fast and accurate short read alignment with Burrows–Wheeler transform. Bioinformatics 2009;25:1754–1760.
[36] Huerta-Cepas J, Forslund K, Coelho LP, et al. Fast Genome-wide functional annotation through orthology assignment by eggNOG-Mapper. Mol Biol Evol 2017;34:2115–2122.
[37] Gffquant (v2.9.1), https://github.com/cschu/gff_quantifier.
[38] **Beghini F**, **McIver LJ**, Blanco-Míguez A, et al. Integrating taxonomic, functional, and strain-level profiling of diverse microbial communities with bioBakery 3. eLife 2021;10:e65088.
[39] Schudoma C, Podlesny D. grp-bork/nHUMAnN: v0.9.1. 2024. https://doi.org/10.5281/zenodo.13143261. Zenodo.
[40] **Schmidt TSB**, **Fullam A**, Feretti P, et al. SPIRE: a searchable, planetary-scale mIcrobiome REsource. Nucleic Acids Res 2024;52:D777–D783.

[41] Benjamini Y, Hochberg Y. Controlling the false discovery rate: a practical and powerful approach to multiple testing. J R Stat Soc Ser B (Methodol) 1995;57:289–300.

[42] Gåfvels M, Olin M, Chowdry BP, et al. Structure and chromosomal assignment of the sterol 12alpha-hydroxylase gene (CYP8B1) in human and mouse: eukaryotic cytochrome P-450 gene devoid of introns. Genomics 1999;56:184–196.

[43] Chen M, Drury JE, Penning TM. Substrate specificity and inhibitor analyses of human steroid 5β-reductase (AKR1D1). Steroids 2011;76:484–490.

[44] Kullak-Ublick GA, Ismair MG, Stieger B, et al. Organic anion-transporting polypeptide B (OATP-B) and its functional comparison with three other OATPs of human liver. Gastroenterology 2001;120:525–533.

[45] Morita S, Kobayashi A, Takanezawa Y, et al. Bile salt-dependent efflux of cellular phospholipids mediated by ATP binding cassette protein B4. Hepatology 2007;46:188–199.

[46] Doden HL, Ridlon JM. Microbial hydroxysteroid dehydrogenases: from alpha to omega. Microorganisms 2021;9:469.

[47] Foley MH, O'Flaherty S, Barrangou R, et al. Bile salt hydrolases: gatekeepers of bile acid metabolism and host-microbiome crosstalk in the gastrointestinal tract. PLOS Pathog 2019;15:e1007581.

[48] Song Z, Cai Y, Lao X, et al. Taxonomic profiling and populational patterns of bacterial bile salt hydrolase (BSH) genes based on worldwide human gut microbiome. Microbiome 2019;7:9.

[49] Ridlon JM, Kang D-J, Hylemon PB. Isolation and characterization of a bile acid inducible 7α-dehydroxylating operon in *Clostridium hylemonae* TN271. Anaerobe 2010;16:137–146.

[50] **Yang Y**, **Wenxing G**, Zhu R, et al. Systematic identification of secondary bile acid production genes in global microbiome. mSystems 2025;10:e00817-24.

[51] Heinken A, Ravcheev DA, Baldini F, et al. Systematic assessment of secondary bile acid metabolism in gut microbes reveals distinct metabolic capabilities in inflammatory bowel disease. Microbiome 2019;7:75.

[52] Harris SC, Devendran S, Alves JMP, et al. Identification of a gene encoding a flavoprotein involved in bile acid metabolism by the human gut bacterium *Clostridium scindens* ATCC 35704. Biochim Biophys Acta Mol Cell Biol Lipids 2018;1863:276–283.

[53] Wise JL, Cummings BP. The 7-α-dehydroxylation pathway: an integral component of gut bacterial bile acid metabolism and potential therapeutic target. Front Microbiol 2023;13:1093420.

[54] Vital M, Rud T, Rath S, et al. Diversity of bacteria exhibiting bile acid-inducible 7α-dehydroxylation genes in the human gut. Comput Struct Biotechnol J 2019;17:1016–1019.

[55] Jin M, Kalainy S, Baskota N, et al. Faecal microbiota from patients with cirrhosis has a low capacity to ferment non-digestible carbohydrates into short-chain fatty acids. Liver Int 2019;39:1437–1447.

[56] Lepercq P, Gerard P, Beguet F, et al. Isolates from normal human intestinal flora but not lactic acid bacteria exhibit 7α- and 7β-hydroxysteroid dehydrogenase activities. Microb Ecol Health Dis 2004;16:195–201.

[57] **Liu C**, **Meng-Xuan D**, **Rexiding A**, et al. Enlightening the taxonomy darkness of human gut microbiomes with a cultured biobank. Microbiome 2021;9:119.

[58] Zhong S, Chèvre R, Castaño Mayan D, et al. Haploinsufficiency of *CYP8B1* associates with increased insulin sensitivity in humans. J Clin Invest 2022;132:e152961.

[59] Lin RC, Fillenwarth MJ, Du X. Cytotoxic effect of 7alpha-hydroxy-4-cholesten-3-one on HepG2 cells: hypothetical role of acetaldehyde-modified delta4-3-ketosteroid-5beta-reductase (the 37-kd-liver protein) in the pathogenesis of alcoholic liver injury in the rat. Hepatology 1998;27:100–107.

[60] Nikolaou N, Gathercole LL, Marchand L, et al. AKR1D1 is a novel regulator of metabolic phenotype in human hepatocytes and is dysregulated in non-alcoholic fatty liver disease. Metabolism 2019;99:67–80.

[61] **Sun L**, **Xie C**, **Wang G**, et al. Gut microbiota and intestinal FXR mediate the clinical benefits of metformin. Nat Med 2018;24:1919–1929.

[62] Yang J, Tang X, Liang Z, et al. Taurocholic acid promotes hepatic stellate cell activation via S1PR2/p38 MAPK/YAP signaling under cholestatic conditions. CMH 2023;29:465–481.

[63] Engen PA, Green SJ, Voigt RM, et al. The gastrointestinal microbiome. Alcohol Res 2015;37:223–236.

[64] **Israelsen M**, **Alvarez-Silva C**, Madsen BS, et al. Impact of acute alcohol consumption on circulating microbiome in asymptomatic alcohol-related liver disease. Gut 2024;73:1041–1044.

[65] Trebicka J, Bork P, Krag A, et al. Utilizing the gut microbiome in decompensated cirrhosis and acute-on-chronic liver failure. Nat Rev Gastroenterol Hepatol 2021;18:167–180.

[66] Slijepcevic D, Kaufman C, Wichers CGK, et al. Impaired uptake of conjugated bile acids and hepatitis b virus pres1-binding in Na+-taurocholate cotransporting polypeptide knockout mice. Hepatology 2015;62:207.

[67] Dawson PA. Hepatic bile acid uptake in humans and mice: multiple pathways and expanding potential role for gut-liver signaling. Hepatology 2017;66:1384–1386.

[68] Weigand K, Köninger J, Encke J, et al. Acute cholecystitis - early laparoskopic surgery versus antibiotic therapy and delayed elective cholecystectomy: ACDC-study. Trials 2007;8:29.

[69] Stanca C, Jung D, Meier PJ, et al. Hepatocellular transport proteins and their role in liver disease. World J Gastroenterol 2001;7:157–169.

[70] Matye DJ, Li Y, Chen C, et al. Gut-restricted apical sodium-dependent bile acid transporter inhibitor attenuates alcohol-induced liver steatosis and injury in mice. Alcohol Clin Exp Res 2021;45:1188–1199.

[71] Hou L, Yang Le, Na Chang, et al. Macrophage sphingosine 1-phosphate receptor 2 blockade attenuates liver inflammation and fibrogenesis triggered by NLRP3 inflammasome. Front Immunol 2020;11:1149.

[72] Wang Y, Aoki H, Yang J, et al. The role of sphingosine 1-phosphate receptor 2 in bile-acid–induced cholangiocyte proliferation and cholestasis-induced liver injury in mice. Hepatology 2017;65:2005–2018.

[73] Stieger B, Meier Y, Meier PJ. The bile salt export pump. Pflugers Arch Eur J Physiol 2007;453:611–620.

[74] Rodrigues AD, Lai Y, Cvijic ME, et al. Drug-induced perturbations of the bile acid pool, cholestasis, and hepatotoxicity: mechanistic considerations beyond the direct inhibition of the bile salt export pump. Drug Metab Dispos 2014;42:566–574.

[75] Stenman LK, Holma R, Eggert A, et al. A novel mechanism for gut barrier dysfunction by dietary fat: epithelial disruption by hydrophobic bile acids. Am J Physiol Gastrointest Liver Physiol 2013;304:G227–G234.

[76] Bernstein C, Holubec H, Bhattacharyya AK, et al. Carcinogenicity of deoxycholate, a secondary bile acid. Arch Toxicol 2011;85:863–871.

[77] Sokol RJ, Dahl R, Devereaux MW, et al. Human hepatic mitochondria generate reactive oxygen species and undergo the permeability transition in response to hydrophobic bile acids. J Pediatr Gastroenterol Nutr 2005;41:235–243.

[78] Martínez-Augustin O, de Medina FS. Intestinal bile acid physiology and pathophysiology. World J Gastroenterol 2008;14:5630–5640.

[79] Way GW, Jackson KG, Muscu SR, et al. Key signaling in alcohol-associated liver disease: the role of bile acids. Cells 2022;11:1374.

[80] Kowdley KV. Ursodeoxycholic acid therapy in hepatobiliary disease. Am J Med 2000;108:481–486.

[81] Perez M-J, Briz O. Bile-acid-induced cell injury and protection. World J Gastroenterol 2009;15:1677–1689.

[82] Litvinov D, Selvarajan K, Garelnabi M, et al. Anti-atherosclerotic actions of azelaic acid, an end product of linoleic acid peroxidation, in mice. Atherosclerosis 2010;209:449–454.

[83] Muthulakshmi S, Saravanan R. Protective effects of azelaic acid against high-fat diet-induced oxidative stress in liver, kidney and heart of C57BL/6J mice. Mol Cell Biochem 2013;377:23–33.

Keywords: Steatotic liver disease (SLD); Enterohepatic circulation; Gut microbiome; Gut–liver axis.

Received 3 January 2026; received in revised form 25 March 2026; accepted 27 March 2026; Available online 2 April 2026

Journal of Hepatology, Volume *8*

Supplemental information

Alcohol-related liver disease disrupts bile acid homeostasis and gut microbial bile acid metabolism

Marisa Isabell Keller, Andressa de Zawadzki, Maja Thiele, Tommi Suvitaival, Karolina Sulek, Michael Kuhn, Christian Schudoma, Daniel Podlesny, Suguru Nishijima, Anthony Fullam, Chan Yeong Kim, Lili Niu, Asger Wretlind, Johanne Krag Hansen, Mads Israelsen, Stine Johansen, Wasiu Akanni, Diënty Hazenbrink, Helene Baek Juel, Matthias Mann, Torben Hansen, Aleksander Krag, Peer Bork, Cristina Legido-Quigley, and on behalf of GALAXY & MicrobLiver consortia

Alcohol-related liver disease disrupts bile acid homeostasis and gut microbial bile acid metabolism

Marisa Isabell Keller, Andressa de Zawadzki, Maja Thiele, Tommi Suvitaival, Karolina Sulek, Michael Kuhn, Christian Schudoma, Daniel Podlesny, Suguru Nishijima, Anthony Fullam, Chan Yeong Kim, Lili Niu, Asger Wretlind, Johanne Krag Hansen, Mads Israelsen, Stine Johansen, Wasiu Akanni, Diënty Hazenbrink, Helene Baek Juel, Matthias Mann, Torben Hansen, Aleksander Krag, Peer Bork, Cristina Legido-Quigley, on behalf of GALAXY & MicrobLiver consortia

Table of contents

Fig. S1 2
Fig. S2 3
Fig. S3 4
Fig. S4 5
Fig. S5 6
Fig. S6 7
Fig. S7 8
Fig. S8 9
Fig. S9 10
Fig. S10 11
Fig. S11 12
Table S1 13

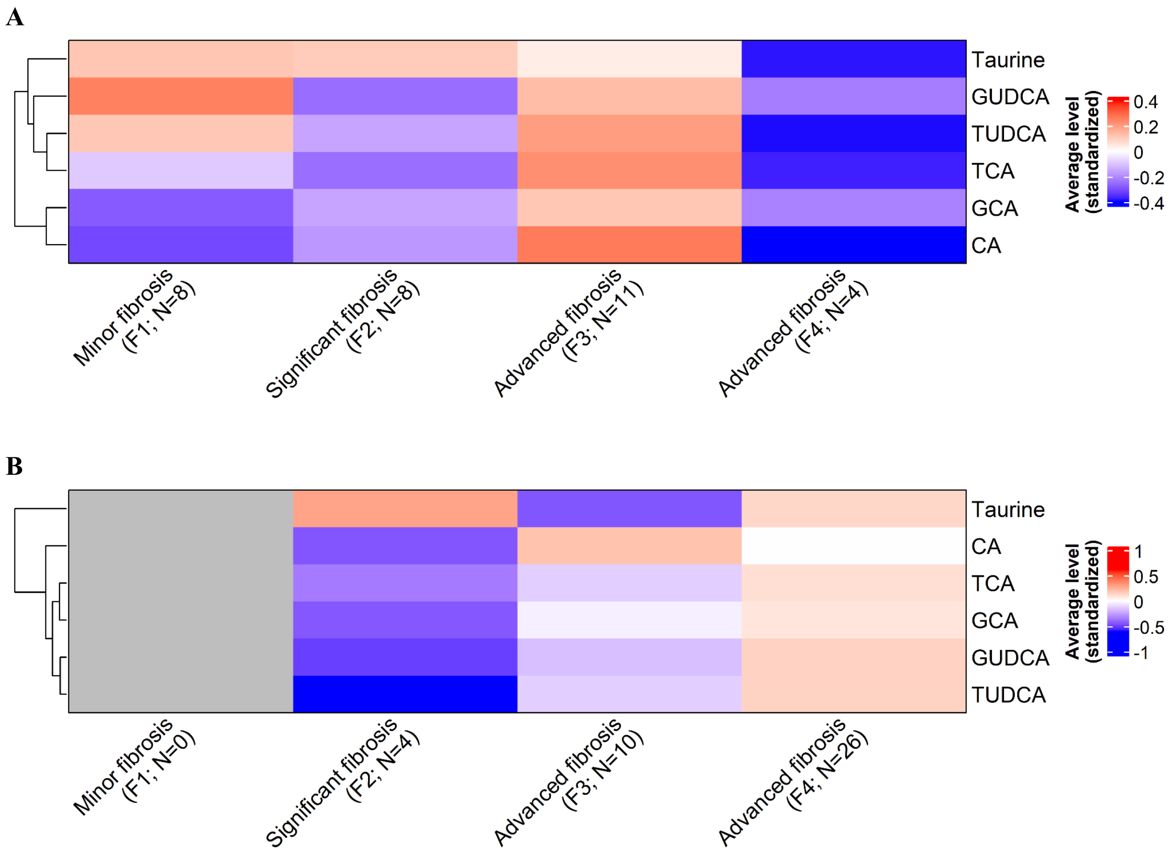

Fig. S1

Blood metabolite levels according to fibrosis stage in the validation cohorts 1 and 2 (A and B, respectively).

Metabolites are shown in rows (ordered by hierarchical clustering), fibrosis stages in columns, and the respective standardized average level in color (red: above cohort average; blue: below cohort average).

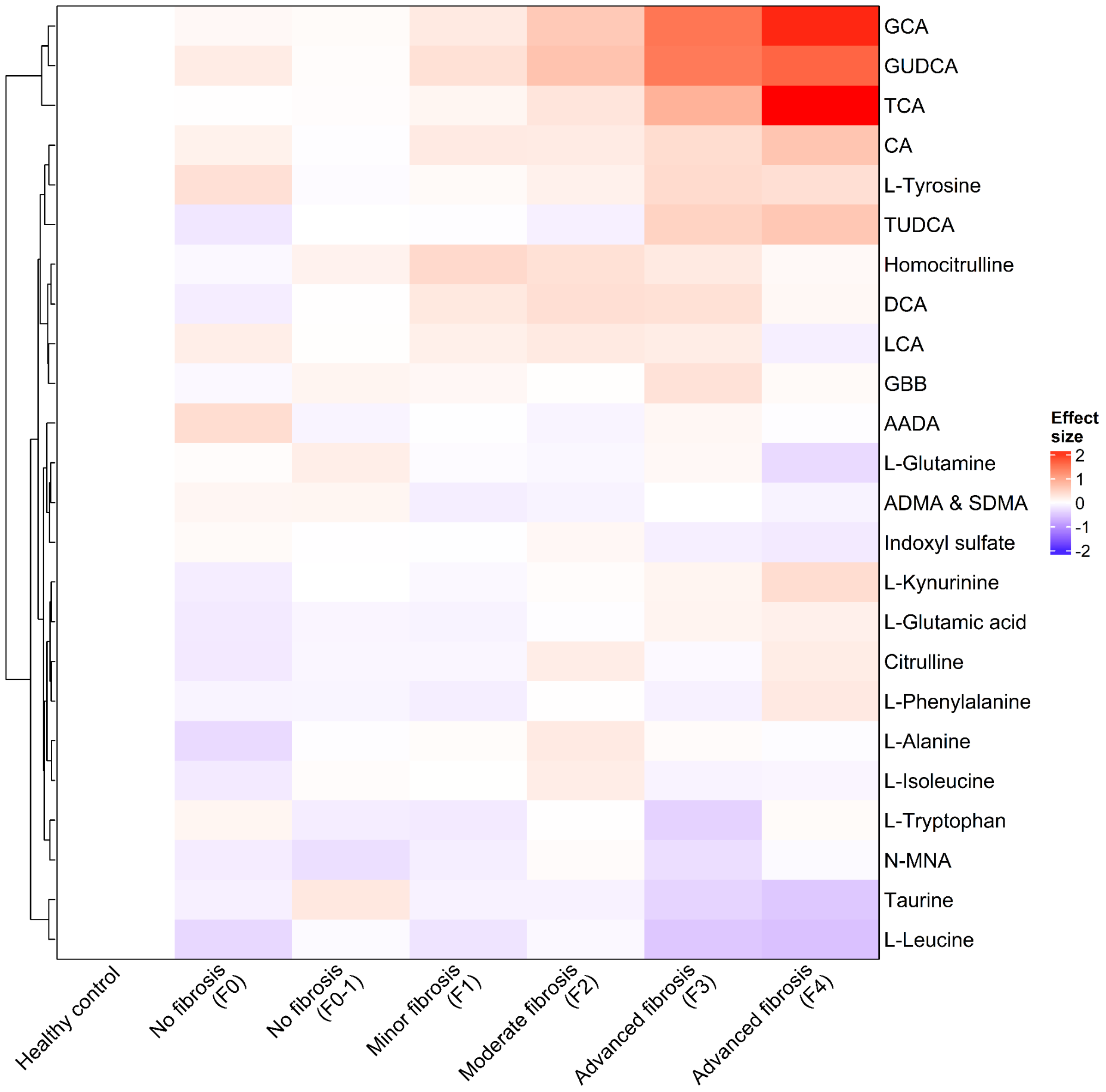

Fig. S2

Blood metabolite levels according to fibrosis stage. Heatmap of the standardized effect size of metabolite level as compared to healthy controls with all measured metabolites in blood. Metabolites are shown in rows (ordered by hierarchical clustering), fibrosis stages in columns, and the respective effect size in color (red: increase; blue: decrease). **Amino acids**: Citrulline, L-alanine, L-glutamic acid, L-glutamine, L-isoleucine, L-leucine, L-phenylalanine, L-tryptophan, L-tyrosine; **Amino acid derivatives**: Asymmetric dimethylarginine and symmetric dimethylarginine (ADMA & SDMA), homocitrulline, L-kynurinine, gamma-butyrobetaine (GBB), taurine; **Primary bile acids**: Cholic acid (CA), chenodeoxycholic acid (CDCA); **Secondary bile acids**: Deoxycholic acid (DCA), lithocholic acid (LCA); **Conjugated primary bile acids**: glycocholic acid (GCA), glycochenodeoxycholic acid (GCDCA), taurocholic acid (TCA), taurochenodeoxycholic acid (TCDCA);

Conjugated secondary bile acids: Glycodeoxycholic acid (CDCA), glycolithocholic acid (GLCA), glycoursodeoxycholic acid (GUDCA), taurodeoxycholic acid (TDCA), tauroursodeoxycholic acid (TUDCA); **Other compounds**: arylacetamide deacetylase (AADA), azelaic acid, N-1-methylnicotinamide (N-MNA), indoxyl sulfate

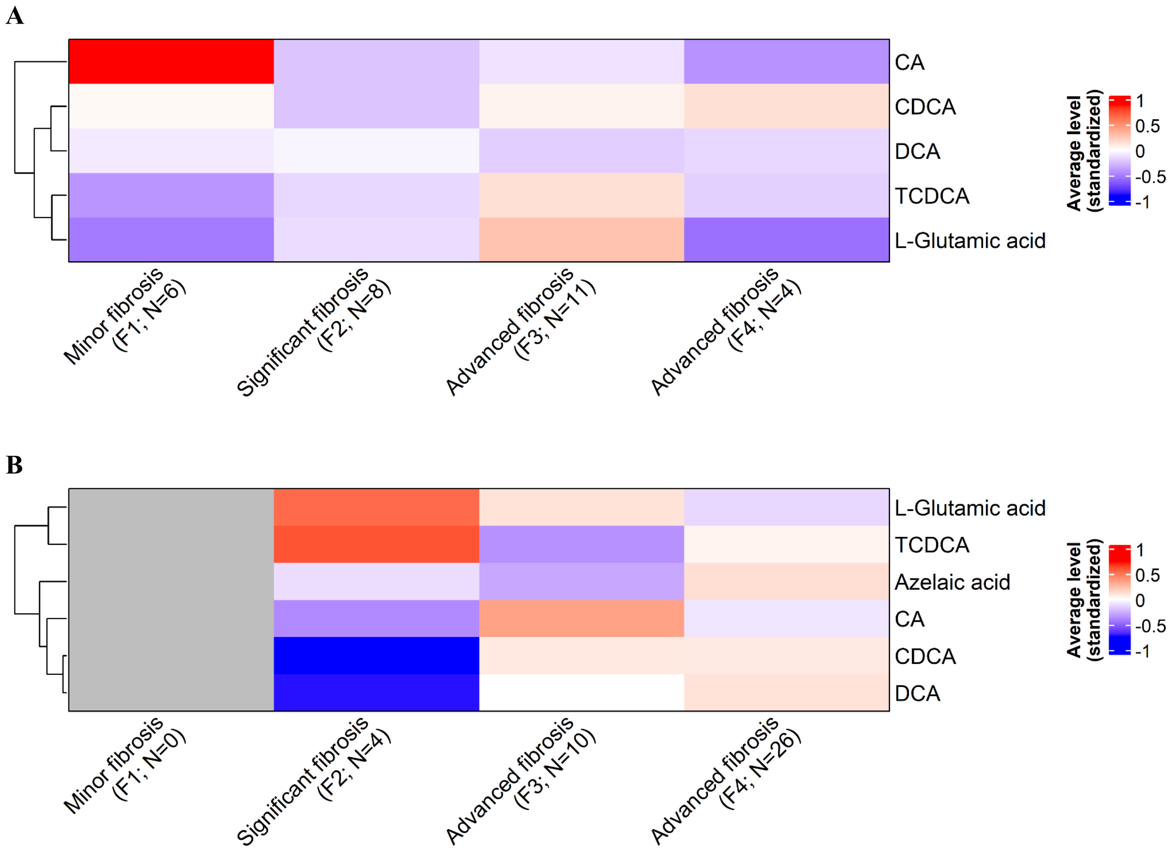

Fig. S3

Fecal metabolite levels according to fibrosis stage in the validation cohorts 1 and 2 (A and B, respectively). Metabolites are shown in rows (ordered by hierarchical clustering), fibrosis stages in columns, and the respective standardized average level in color (red: above cohort average; blue: below cohort average).

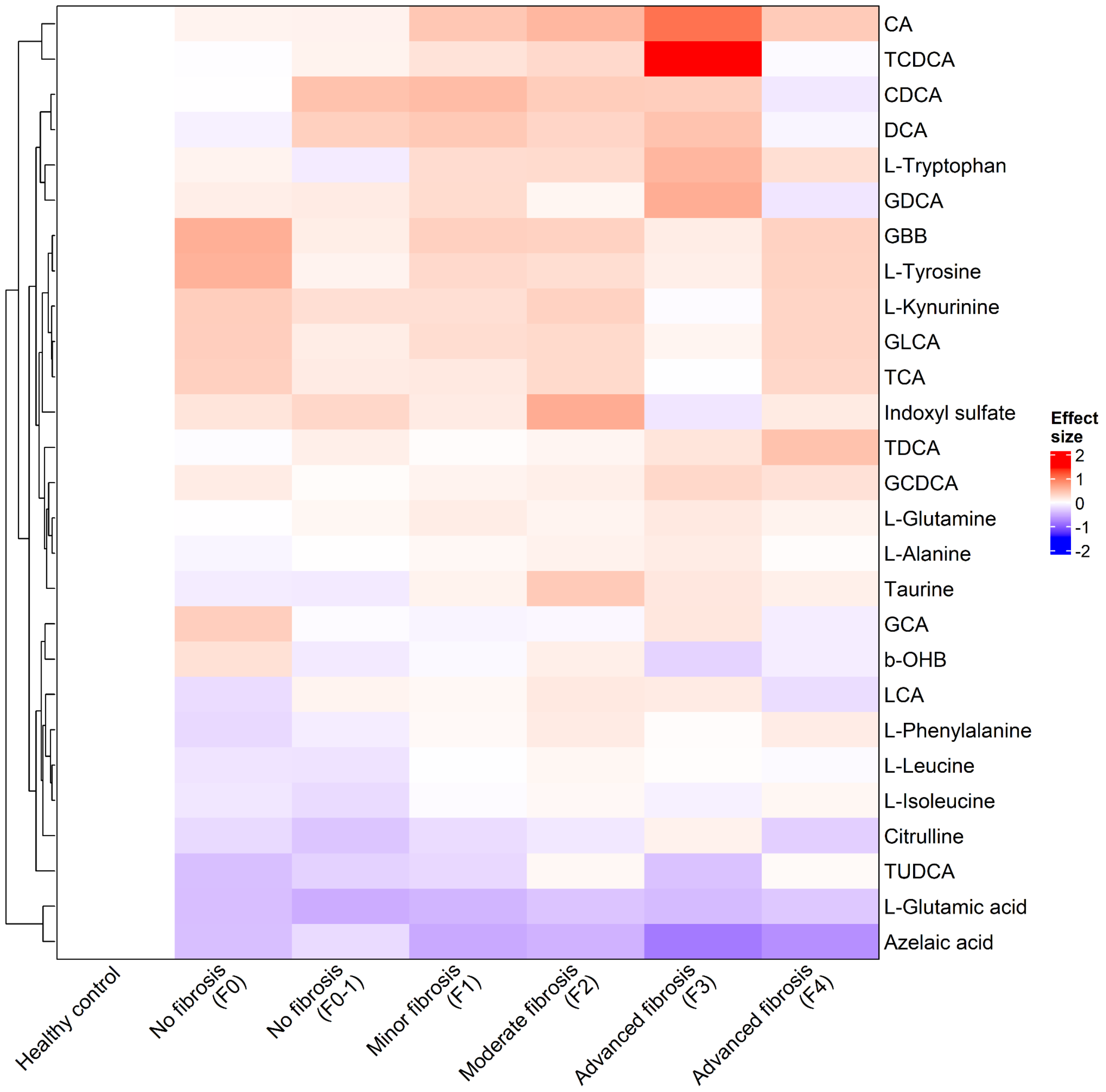

Fig. S4

Fecal metabolite levels according to fibrosis stage. Heatmap of the standardized effect size of metabolite level as compared to healthy controls with all measured metabolites in feces. Metabolites are shown in rows (ordered by hierarchical clustering), fibrosis stages in columns, and the respective effect size in color (red: increase; blue: decrease). **Amino acids**: Citrulline, L-alanine, L-glutamic acid, L-glutamine, L-isoleucine, L-leucine, L-phenylalanine, L-tryptophan, L-tyrosine; **Amino acid derivatives**: Asymmetric dimethylarginine and symmetric dimethylarginine (ADMA & SDMA), homocitrulline, L-kynurinine, gamma-butyrobetaine (GBB), taurine; **Primary bile acids**: Cholic acid (CA), chenodeoxycholic acid (CDCA); **Secondary bile acids**: Deoxycholic acid (DCA), lithocholic acid (LCA); **Conjugated primary bile acids**: glycocholic acid (GCA), glycochenodeoxycholic acid (GCDCA), taurocholic acid (TCA), taurochenodeoxycholic acid (TCDCA);

Conjugated secondary bile acids: Glycodeoxycholic acid (CDCA), glycolithocholic acid (GLCA), glycoursodeoxycholic acid (GUDCA), taurodeoxycholic acid (TDCA), tauroursodeoxycholic acid (TUDCA); **Other compounds**: arylacetamide deacetylase (AADA), azelaic acid, N-1-methylnicotinamide (N-MNA), indoxyl sulfate

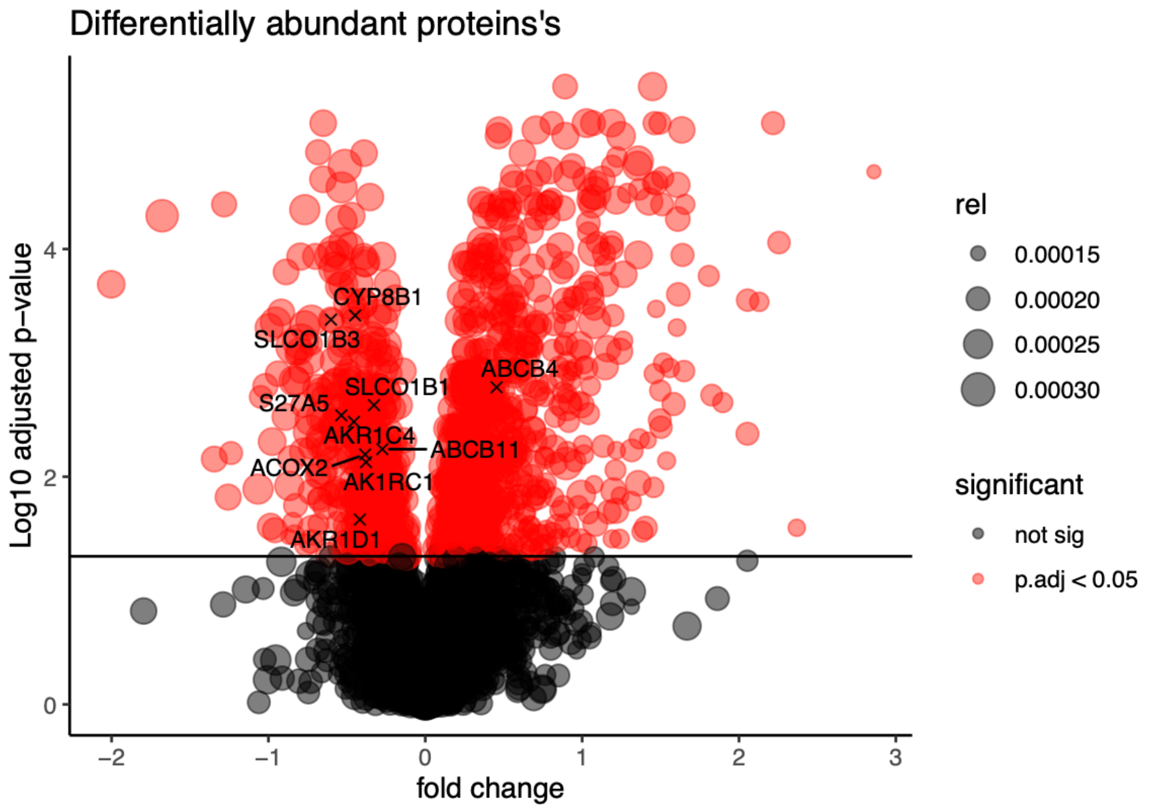

Fig. S5

Differential abundance analysis of the liver proteomics dataset comparing early fibrosis (F0-1) with late fibrosis (F2-4). Proteins in red are significantly expressed after correcting for false positives using the Benjamini-Hochberg correction. Points with labels are related to BA synthesis and transport.

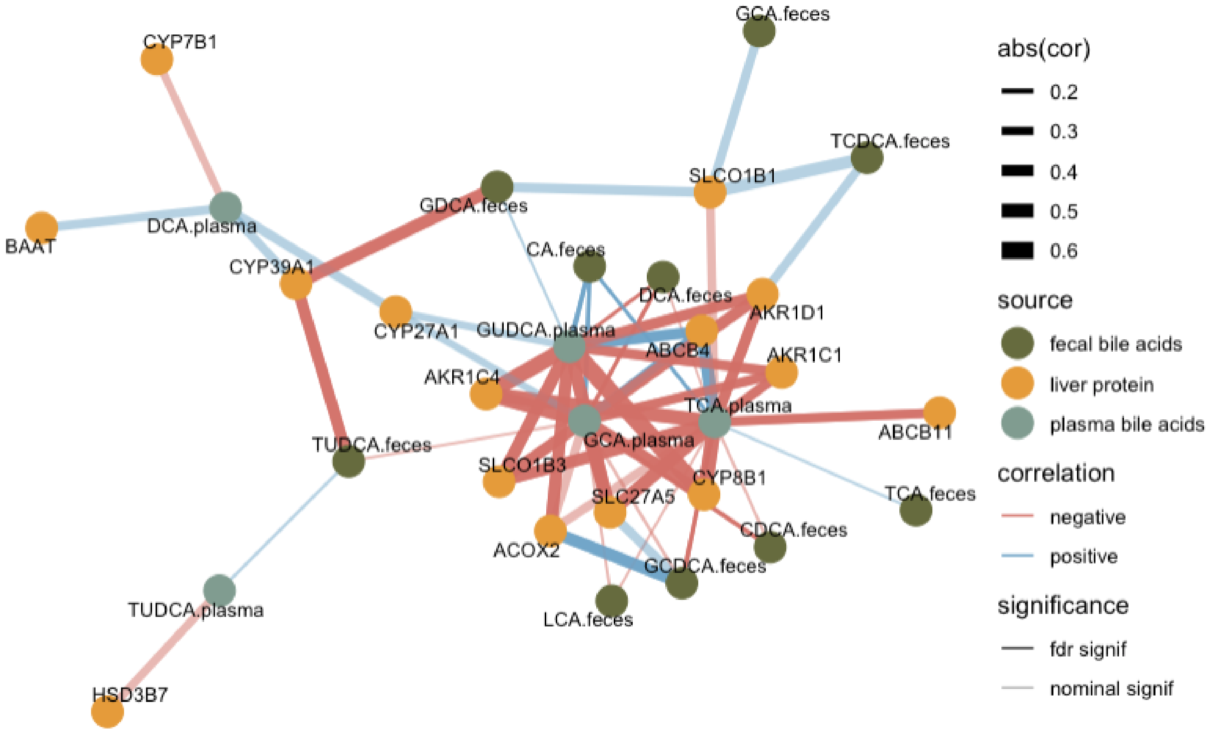

Fig. S6

Network analysis of liver proteins related to bile acid metabolism and bile acids measured in feces and plasma. The network is based on Spearman correlations, and "cor" represents the absolute value of the Spearman ρ. Shown are only the correlations with nominal significance and correlations with FDR-corrected significance, when correcting for all correlations performed in this study (including plasma bile acids, fecal bile acids, liver proteins, microbial abundance, and microbial gene abundances).

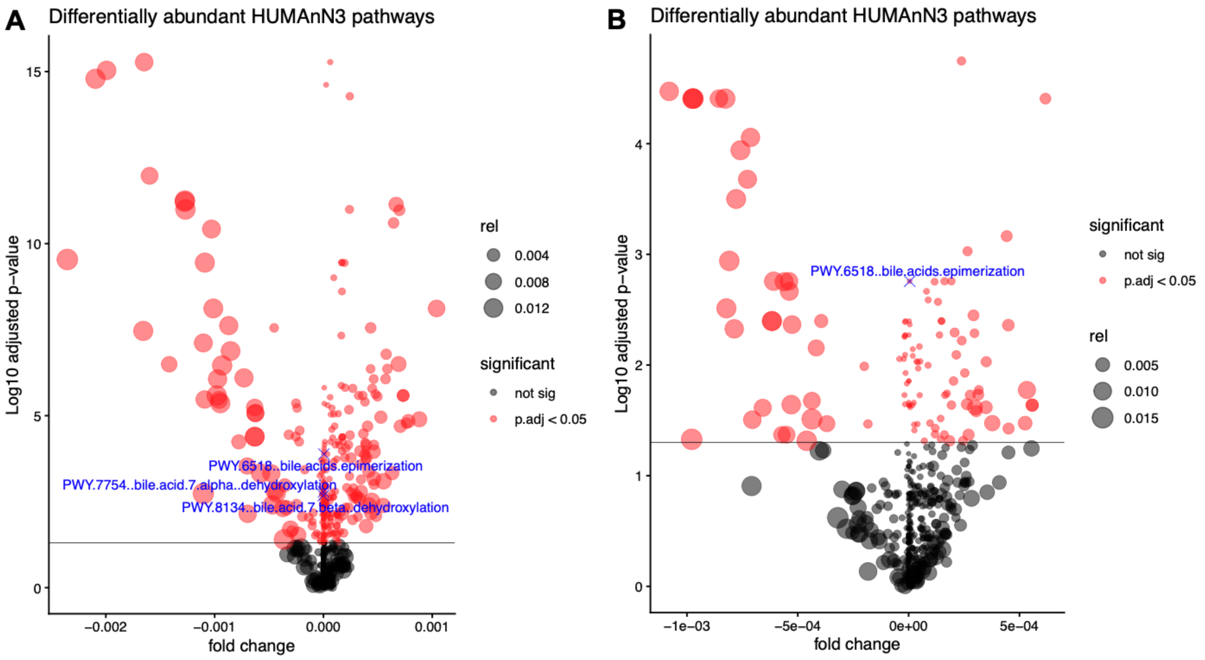

Fig. S7

Differential abundance analysis of the microbial pathways derived from metagenomic sequencing of fecal samples. The test compares the ALD with the HC samples (A) and within the ALD cohort, the early fibrosis (F0-1) with late fibrosis (F2-4) (B). Points in red are significantly changing pathways between the comparisons after adjusting for false positives using the Benjamini-Hochberg correction. Points with labels show secondary BA metabolism pathways.

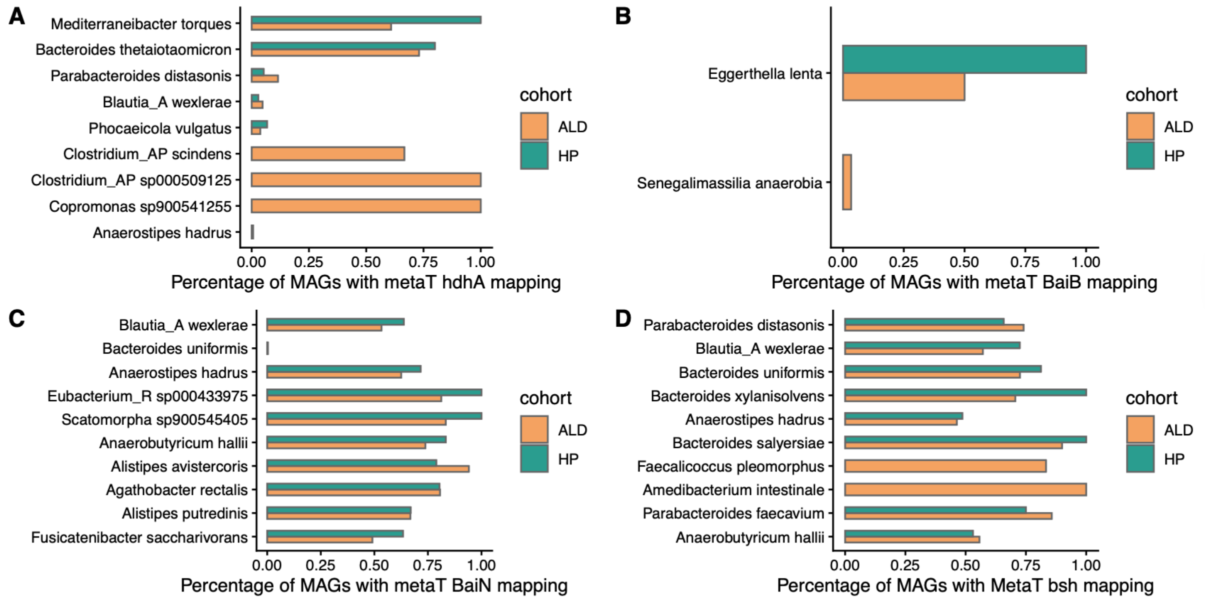

Fig. S8

Mapping of the metatranscriptomic reads assigned to the KEGG orthologs to the MAGs within the cohort. The fraction of MAGs is calculated by dividing the species-specific MAGs with mapping for this gene by the total amount of species-specific MAGs. The species are selected to correspond to the species reported in figure 5 and Fig. S6. hdhA = K00076, BaiB = K15868, BaiN = K07007, bsh = K01442

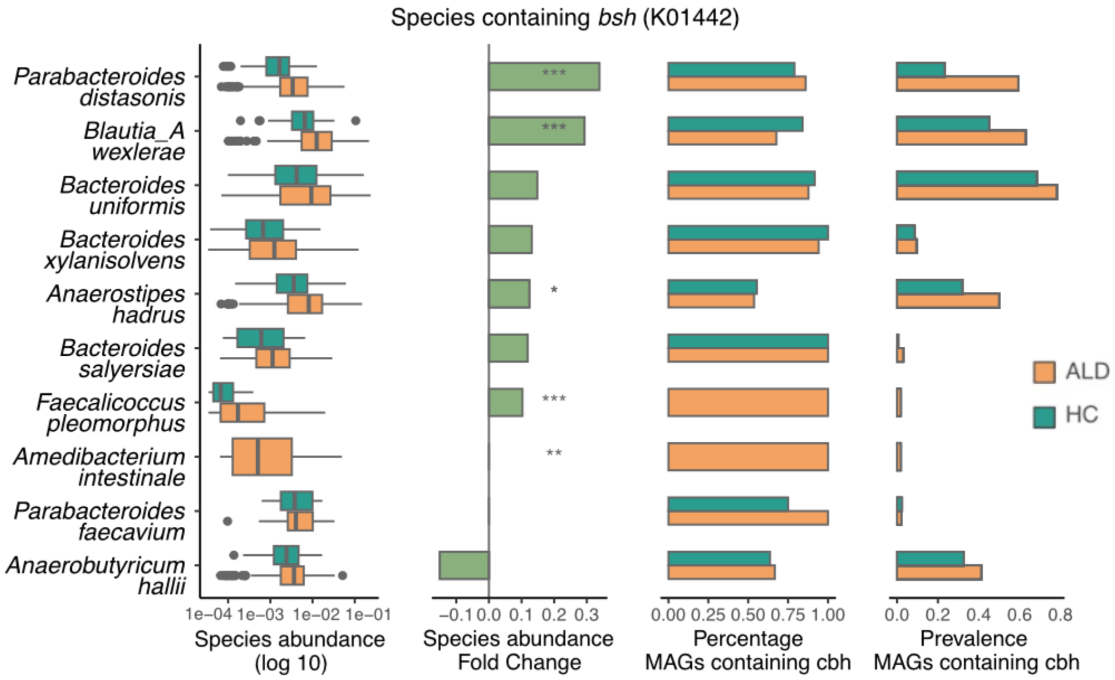

Fig. S9

Microbial taxa for the secondary bile acid metabolism deconjugation pathway facilitated by *bsh*. The species abundance, species fold change between healthy and ALD samples, the gene-carrying frequency of MAGs, and the prevalence of gene-carrying MAGs in the cohort collectively estimate the importance of the taxa in ALD.

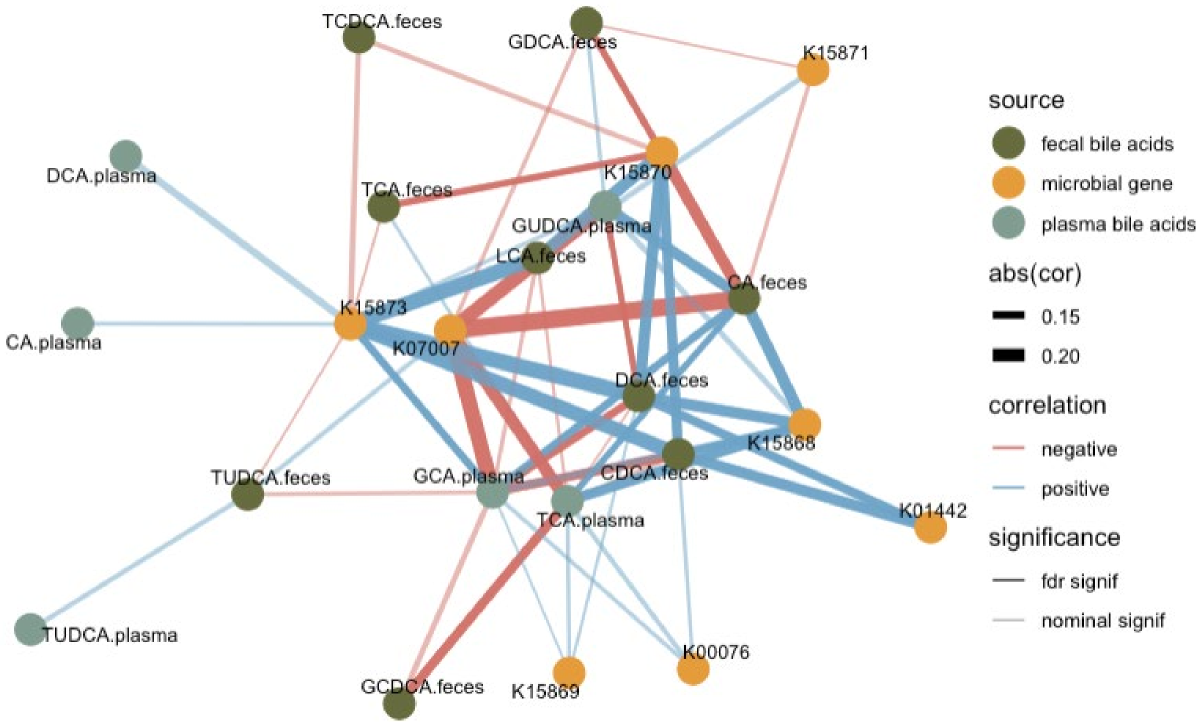

Fig. S10

Network analysis of microbial bile-acid metabolism-related KOs and bile acids measured in feces and plasma. The network is based on Spearman correlations, and "cor" represents the absolute value of the Spearman ρ. Shown are only the correlations with nominal significance and correlations with FDR-corrected significance, when correcting for all correlations performed in this study (including plasma bile acids, fecal bile acids, liver proteins, microbial abundance, and microbial gene abundances). K07007 = BaiN, K00076 = hdhA, K01442 = bile-salt-hydrolase (bsh), K15868 = BaiB, K15869 = BaiA, K15870 = BaiCD, K15871 = BaiF, K15873 = BaiH.

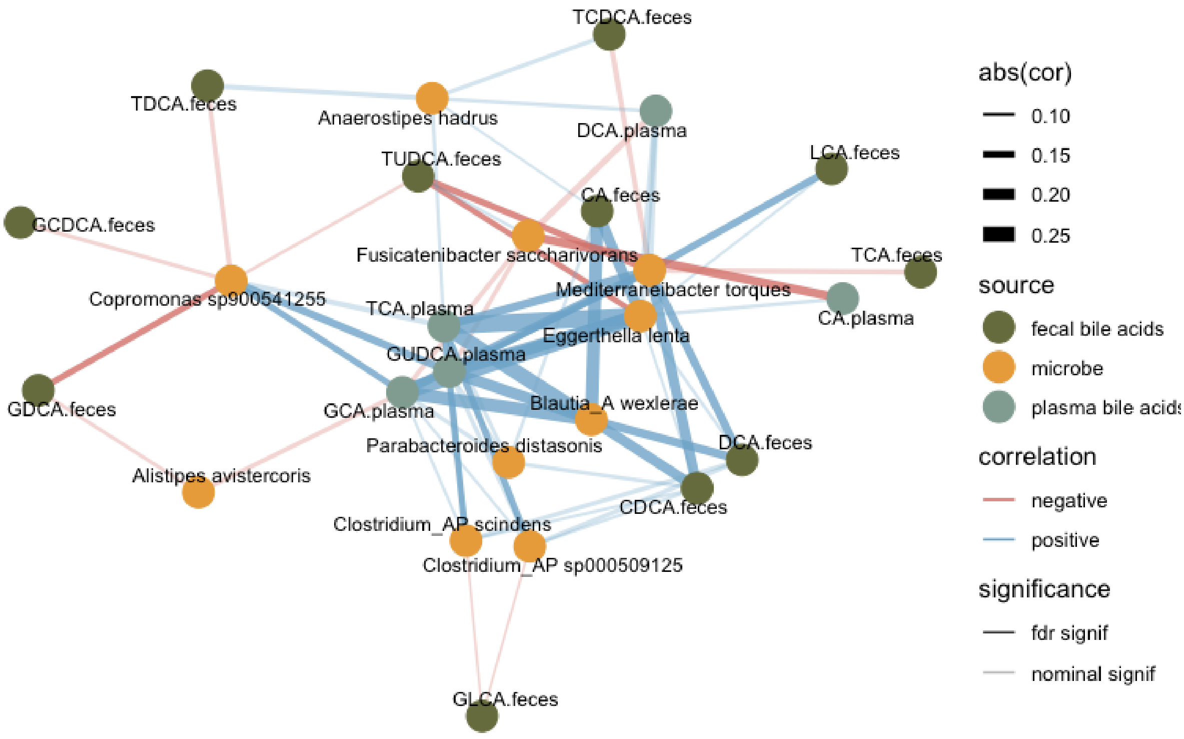

Fig. S11

Network analysis of microbial abundances and bile acids measured in feces and plasma. The network is based on Spearman correlations and “cor” represents the absolute value of the Spearman ρ. Shown are only the correlations with nominal significance and correlations with FDR-corrected significance, when correcting for all correlations performed in this study (including plasma bile acids, fecal bile acids, liver proteins, microbial abundance, and microbial gene abundances). Shown are only the microbes that we identified as relevant in our study based on the gene mappings to their MAGs. But all microbes with a mean abundance higher than 1e-4 and a prevalence of more than 10% of the samples have been included in the correlation analysis.

	GALA-ALD	Validation cohort 1	Validation cohort 2	p-value
**Characteristics**				
Participants (n)	462	34	52	NA
Female sex (n, %)	112(24.2%)	2(5.9%)	14(26.9%)	0.0371
Age (years)	56.5 ± 10.4	59.2 ± 8.7	60.7 ± 7.9	0.0122
BMI (kg/m^2)	27.5 ± 5.3	30.5 ± 5.3	29 ± 5.4	0.0006
T2D (n, %)	64(13.9%)	0(0%)	2(3.8%)	0.0431
Metabolic syndrome (n, %)	114(24.7%)	14(41.2%)	20(38.5%)	0.0650
Abstinent (n, %)	193(41.8%)	30(88.2%)	17(32.7%)	0.0000
**Liver parameter**				
Cap Value (dB/m)	284.8 ± 61.7	306.4 ± 61	288.8 ± 67.4	0.0000
Kleiner Score (0/0-1/1/2/3/4)	36/98/127/107/27/66	0/0/8/8/11/4	0/0/0/5/10/29	0.0000
Bilirubin (umol/L)	12.6 ± 9.5	9 ± 4.7	15.8 ± 9.5	0.0002
ALT (U/L)	39.4 ± 29.5	46.5 ± 41	33.7 ± 30.8	0.0089
AST (U/L)	45.5 ± 35.2	NaN ± NA	42.4 ± 25	0.9551
GGT (U/L)	182.6 ± 315.8	162.9 ± 155.7	180 ± 183.7	0.0515
Platelets (10^9/L)	237 ± 87.6	212.8 ± 51.5	168.1 ± 64	0.0000
MELD-score	7.1 ± 1.8	7.8 ± 1.5	8.7 ± 1.6	0.0000

Table S1

Characteristics of the GALAXY alcohol-related liver disease (ALD) cohort in comparison to the Validation 1 and 2 cohorts. T2D = Type 2 Diabetes, Cap = Controlled attenuation parameter, ALT = alanine transaminase, AST = aspartate aminotransferase, GGT = gamma-glutamyl transferase, MELD = model of end-stage liver disease. The continuous variables are presented as means with standard deviations. P-values report the significance of the differences between the ALD and HC cohorts tested by the Kruskal-Wallis test for continuous variables and the chi-squared test for categorical variables.
